# An Engineered Heterostructured Trinity Enables Fire-Safe, Thermally Conductive Polymer Nanocomposite Films with Low Dielectric Loss

**DOI:** 10.1007/s40820-025-01681-9

**Published:** 2025-02-26

**Authors:** Qiang Chen, Jiabing Feng, Yijiao Xue, Siqi Huo, Toan Dinh, Hang Xu, Yongqian Shi, Jiefeng Gao, Long-Cheng Tang, Guobo Huang, Weiwei Lei, Pingan Song

**Affiliations:** 1 Key Laboratory of Integrated Regulation and Resource Development On Shallow Lakes, Ministry of Education, College of Environment, Hohai University, Nanjing, 210098 People’s Republic of China; 2 Suzhou Research Institute, Hohai University, Suzhou, 215100 People’s Republic of China; 3 College of Biological, Chemical Sciences and Engineering, Jiaxing University, Jiaxing, 314001 People’s Republic of China; 4 Institute of Chemical Industry of Forest Products, Chinese Academy of Forestry (CAF), Nanjing, 210042 People’s Republic of China; 5 Centre for Future Materials, School of Engineering, University of Southern Queensland, Springfield, 4300 Australia; 6 College of Environment and Safety Engineering, Fuzhou University, Fuzhou, 350116 People’s Republic of China; 7 School of Chemistry and Chemical Engineering, Yangzhou University, Yangzhou, 225002 People’s Republic of China; 8 Key Laboratory of Organosilicon Chemistry and Material Technology of MoE, College of Material, Chemistry and Chemical Engineering, Hangzhou Normal University, Hangzhou, 311121 People’s Republic of China; 9 School of Pharmaceutical and Chemical Engineering, Taizhou University, Jiaojiang, 318000 People’s Republic of China; 10 School of Science, RMIT University, Melbourne, VIC 3000 Australia; 11 Centre for Future Materials, School of Agriculture and Environmental Science, University of Southern Queensland, Springfield, 4300 Australia

**Keywords:** Bionic strategy, Fluorinated graphene, Flame retardancy, Thermal conductivity, Dielectric constant

## Abstract

To adapt to the trend of increasing miniaturization and high integration of microelectronic equipments, there is a high demand for multifunctional thermally conductive (TC) polymeric films combining excellent flame retardancy and low dielectric constant (*ε*). To date, there have been few successes that achieve such a performance portfolio in polymer films due to their different and even mutually exclusive governing mechanisms. Herein, we propose a trinity strategy for creating a rationally engineered heterostructure nanoadditive (FG@CuP@ZTC) by in situ self-assembly immobilization of copper-phenyl phosphonate (CuP) and zinc-3, 5-diamino-1,2,4-triazole complex (ZTC) onto the fluorinated graphene (FG) surface. Benefiting from the synergistic effects of FG, CuP, and ZTC and the bionic lay-by-lay (LBL) strategy, the as-fabricated waterborne polyurethane (WPU) nanocomposite film with 30 wt% FG@CuP@ZTC exhibits a 55.6% improvement in limiting oxygen index (LOI), 66.0% and 40.5% reductions in peak heat release rate and total heat release, respectively, and 93.3% increase in tensile strength relative to pure WPU film due to the synergistic effects between FG, CuP, and ZTC. Moreover, the WPU nanocomposite film presents a high thermal conductivity (*λ*) of 12.7 W m^−1^ K^−1^ and a low *ε* of 2.92 at 10^6^ Hz. This work provides a commercially viable rational design strategy to develop high-performance multifunctional polymer nanocomposite films, which hold great potential as advanced polymeric thermal dissipators for high-power-density microelectronics.

**Supplementary Information:**

The online version contains supplementary material available at 10.1007/s40820-025-01681-9.

## Highlights

- The as-fabricated waterborne polyurethane (WPU) nanocomposite film exhibits a 55.6% improvement in limiting oxygen index, 66.0% and 40.5% reductions in peak heat release rate and total heat release, respectively, and 93.3% increase in tensile strength relative to pure WPU film.
- The resultant WPU nanocomposite film presents a high thermal conductivity (*λ*) of 12.7 W m^−1^ K^−1^ and a low dielectric constant (*ε*) of 2.92 at 10^6^ Hz.

**Supplementary Information:**

The online version contains supplementary material available at 10.1007/s40820-025-01681-9.

## Graphical abstract

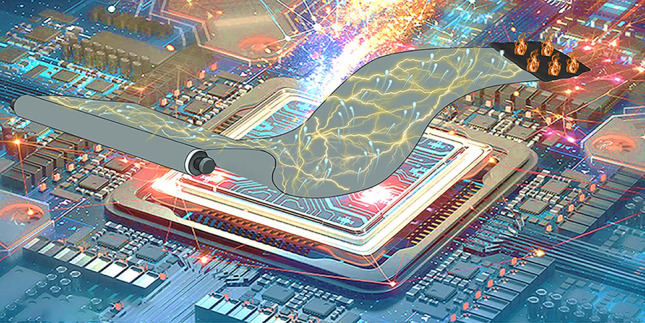

## Introduction

With the mushroom development of fifth-generation (5G) wireless communication systems, internet of things, artificial intelligence, and integrated circuits, the heat dissipation problem has recently became a prominent challenge for the microelectronic devices with their continuous evolution toward high integration, large power, miniaturization, and densification [1–3]. The over-heating caused by heat accumulation will seriously affect the operating efficiency, reliability, and working life of these microelectronic devices and even cause fire [4, 5]. Additionally, the fast signal transmission of these microelectronic devices places higher dielectric property requirements on the substrate materials [6]. In this context, it is highly imperative to design multifunctional microelectronic materials with high thermal conductivity (*λ*), satisfactory fire retardancy, and low dielectric constant (*ε*) for safe and effective heat dissipation without compromising transmission loss [7–9].

Polymer-based thermally conductive (TC) nanocomposite films, as a key cooling material in microelectronic devices, possess good mechanical properties, water resistance, and easy accessibility. To ensure the safe high-efficiency heat dissipation without negative impact on device operation, these polymer nanocomposite TC films generally need to show an integrated performance portfolio—that is, high *λ*, desired fire safety, and low *ε* (or low dielectric loss [10–16]. To date, very few successes have been reported to achieve such a performance portfolio in polymeric materials due to their different and even mutually exclusive governing mechanisms. For example, Zhong et al*.* and He et al., respectively, prepared polyethylene terephthalate (PET) and polydimethylsiloxane (PDMS) nanocomposite materials with a *λ* of 5.37 and 7.31 W m^−1^ K^−1^, respectively [1, 12]. Unfortunately, these *λ* values remain unsatisfactory, in addition to a lack of addressing fire risks of these materials. Dong et al. reported a polyimide (PI) nanocomposite film by the combination of ionic liquids and calcium fluoride nanofillers, which presents a low *ε* of 2.14 at 10^6^ Hz but a still relatively low *λ* of 7.22 W m^−1^ K^−1^ [9]. Further, the existing preparation approaches of these polymer TC materials suffer from some drawbacks, such as an inability to prepare large-scale nanocomposite films, use of a large amount of organic solvents, and complicated preparation processes. Overall, it remains a grand challenge to achieve high *λ*, excellent fire retardancy, and low dielectric loss in polymer TC film materials.

Fluorinated graphene (FG), a derivative of two-dimensional (2D) graphene with an analogous structure to boron nitride nanosheet (BNNS), has attracted tremendous research interest because of its fascinating feature of ultrahigh in-plane *λ* (1800 W m^−1^ K^−1^), ultralow *ε* (~ 1.2), and excellent electrical insulation [17]. Thus, modifying the surface of FG by phosphorus (P)- or/and nitrogen (N)-containing organic flame retardants with organic segments can improve its interfacial compatibility with the polymeric matrix and greatly reduce the thermal resistance and interfacial polarization in the filler/matrix interfacial regions, thereby significantly improving the TC properties and reducing the *ε* of polymeric nanocomposites [18–20]. Furthermore, the resultant polymer nanocomposite films can also achieve remarkably enhanced fire retardancy [21–29].

To address the aforementioned challenge, in this work, we proposed a trinity strategy by rationally engineering a heterostructure, FG@copper-phenyl phosphonate@zinc-3, 5-diamino-1,2,4-triazole complex (FG@CuP@ZTC), which is then used to prepare TC, fire-safe waterborne polyurethane (WPU)-based nanocomposite films with low *ε* via bionic lay-by-lay (LBL) methodology. The synergistic P-N flame-retardant action enhances the flame-retardant efficiency of functionalized FG for the WPU matrix. Meanwhile, the target FG@CuP@ZTC endows WPU nanocomposite films with high *λ* and low *ε*. In addition, such a facile yet scalable design has led to a desirable performance portfolio in the large-size film, which has never been achieved so far. In general, the work provides an innovative strategy for the ease of mass production of multifunctional polymeric thermal management materials combining excellent flame retardancy and low *ε*, thus creating many new opportunities for real-world applications in the areas of electronic, auto, military, and aerospace, etc.

## Experimental Section

### Materials

FG was purchased from Jiangsu XFNANO Mater. Tech. Co. Ltd. (Nanjing, China). Phenylphosphonic acid (PPA, 98.0%), copper sulfate pentahydrate (CuSO_4_·5H_2_O, 98.0%), sodium hydroxide (NaOH, 97%), zinc sulfate heptahydrate (ZnSO_4_·7H_2_O, 99.5%), ethanol (analytical reagent, 95%), 3,5-diamino-1,2,4-triazole (analytical reagent, 98%), and sodium carboxy methyl cellulose (NaCMC, chemical pure) were provided by Macklin Biochemical Co. Ltd. (Shanghai, China). WPU emulsions with a solid content of 33 wt% were purchased from Shenzhen Jitian Chemical Co. Ltd. (Shenzhen, China). Deionized water (H_2_O) was made in the laboratory.

### Preparation of FG@CuP

FG@CuP was synthesized by in situ self-assembly technique. Specifically, 1.5 g of FG, 0.1 g of NaCMC, and 300 mL H_2_O were added to a ball milling tank containing zirconium beads. After 12 h of ball milling at a rotation speed of 1000 rpm, the FG aqueous dispersion was obtained. Afterward, 200 mL PPA neutralized by NaOH (Na-PPA) aqueous solution (0.1 mol L^−1^) was added into the FG dispersion and stirred for 0.5 h. Then, 5.0 g of CuSO_4_·5H_2_O dissolved in 100 mL H_2_O was added into the above FG dispersion/Na-PPA mixture dropwise, with vigorous stirring for 8 h. Subsequently, the precipitates were centrifuged, washed several times with H_2_O, and freeze-dried (pressure: 10 Pa; temperature: -80 °C) for 48 h. Finally, the products were ground and sieved to obtain FG@CuP powders with a weight ratio of FG and CuP *ca*. 1: 1.8. A similar process was conducted to prepare the FG@CuP with a weight ratio of FG and CuP *ca*. 1: 2.

### Synthesis of FG@CuP@ZTC

The ZTC was loaded onto the surface of FG@CuP by in situ self-assembly method. In brief, 5.6 g of FG@CuP (with a weight ratio of FG and CuP *ca*. 1: 1.8), 0.2 g of NaCMC, and 300 mL H_2_O were subjected to ball milling at 1000 rpm for 12 h. After that, the FG@CuP aqueous dispersion was collected. Then, 0.4 g of 3,5-diamino-1,2,4-triazole was placed into 100 mL H_2_O with ultrasonication for 0.5 h, followed by the addition of the FG@CuP aqueous dispersion and stirred for 0.5 h. 100 mL ZnSO_4_·7H_2_O aqueous solution (0.06 mol L^−1^) was added dropwise into the above FG@CuP dispersion with 3,5-diamino-1,2,4-triazole and reacted for another 8 h. Finally, the target FG@CuP@ZTC with a weight ratio of FG, CuP, and ZTC *ca*. 1: 1.8: 0.2 was collected by centrifugation from the slurry, washing with H_2_O, and freeze-drying (pressure: 10 Pa and temperature: -80 °C) for 48 h.

### Fabrication of WPU Nanocomposite Films

WPU nanocomposite films containing different FG@CuP@ZTC contents were fabricated according to the bionic LBL blade coating procedure, typically a predesigned amount of FG@CuP@ZTC in a 20 mL of ethanol as sonicated and stirred at room temperature for 1 h. Then, a certain amount of WPU emulsions were added to the mixture and stirred for 1 h, followed by scraping and drying at 100 °C for 5 min to get WPU/FG@CuP@ZTC film. Subsequently, the WPU/FG@CuP@ZTC suspension was applied to the surface of WPU/FG@CuP@ZTC film and dried again at 100 °C for 5 min. The above bionic LBL operation was repeated until the thickness of the WPU/FG@CuP@ZTC nanocomposite film reached 100 μm. The neat WPU, WPU/FG@CuP, WPU/FG@ZTC, and WPU/FG/CuP/ZTC samples were also prepared by the above method, and the detailed formulations of all samples are presented in Table S1.

## Results and Discussion

### Design and Characterization of WPU/FG@CuP@ZTC Nanocomposite Film

**Fig. 1:**
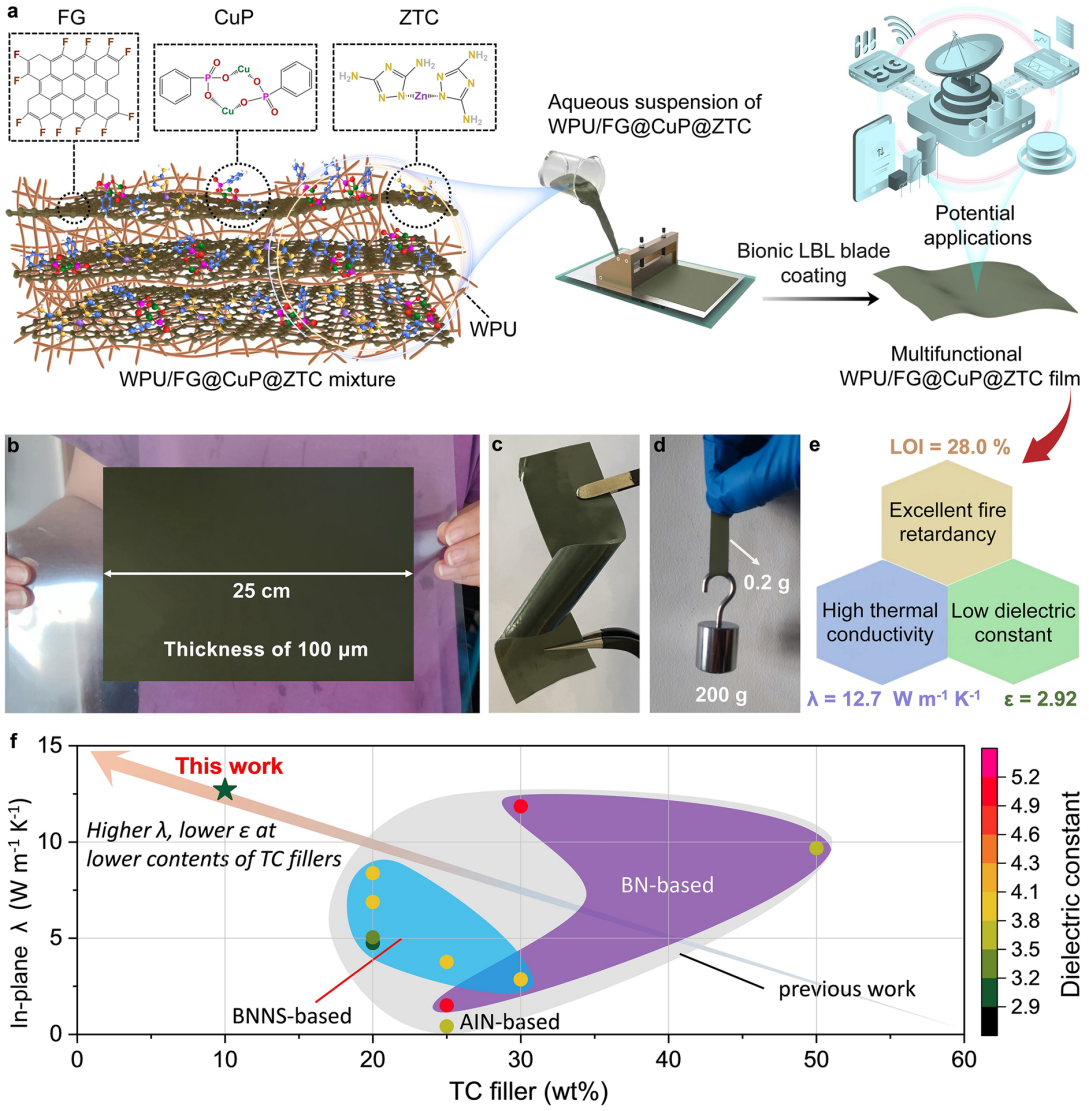
Design and characterization of WPU/FG@CuP@ZTC nanocomposite film. **a** Schematic illustration for the fabrication route of multifunctional WPU/FG@CuP@ZTC nanocomposite film by bionic LBL blade coating procedure. Digital photographs of **b** large-scale WPU/30FG@CuP@ZTC nanocomposite film. **c** WPU/30FG@CuP@ZTC nanocomposite film with ultra-flexibility. **d** WPU/30FG@CuP@ZTC nanocomposite film withstanding a weight of 200 g. **e** Multifunctionality of WPU/30FG@CuP@ZTC nanocomposite film. **f** Comparison of comprehensive performances of WPU/30FG@CuP@ZTC nanocomposite film with other counterparts

The preparation route of multifunctional WPU/FG@CuP@ZTC nanocomposite film is schematically illustrated in Fig. 1a, which involves the in situ growth of CuP and ZTC on the FG surface and the bionic LBL blade coating procedure. Large-scale WPU/30FG@CuP@ZTC nanocomposite film (size: 25 cm × 17 cm, thickness: 100 μm) can be prepared by the bionic LBL blade coating method (Fig. 1b). This WPU nanocomposite film can been easily bent without any damage (Fig. 1c). In addition, the as-prepared WPU/30FG@CuP@ZTC film (0.2 g of weight) can withstand 200 g of weight without cracking or breaking (Fig. 1d). The results indicate that the WPU/30FG@CuP@ZTC nanocomposite film has eminent flexibility and mechanical strength. The as-fabricated WPU/30FG@CuP@ZTC nanocomposite film demonstrates superior versatility, which exhibits a limiting oxygen index (LOI) of 28.0%, a high *λ* of 12.7 W m^−1^ K^−1^, as well as a low *ε* of 2.92 at 10^6^ Hz (see Fig. 1e). Such a desired performance portfolio makes it highly suitable for potential applications in the microelectronic fields, such as 5G system. More importantly, as shown in Fig. 1f, the target WPU/30FG@CuP@ZTC nanocomposite film outperforms existing counterparts (including BNNS-based, BN-based, and AlN-based nanocomposite materials) in the integrated performance portfolio, including higher *λ*, lower *ε* at lower contents of TC fillers, which is yet to be achieved in other nanocomposite films.

### Structural Characterizations of FG@CuP@ZTC

**Fig. 2:**
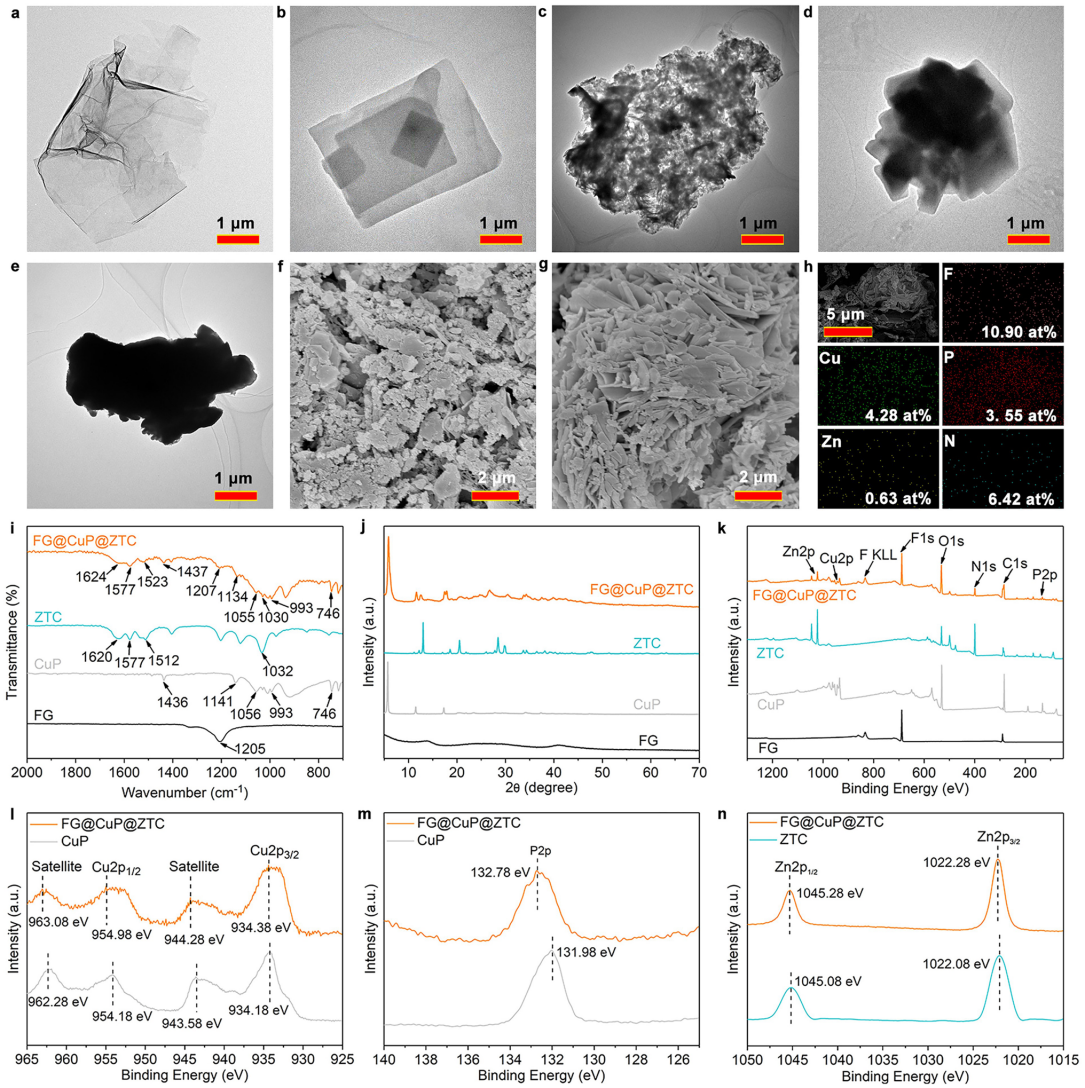
Structural characterizations. TEM images of **a** FG, **b** CuP, **c** FG@CuP, **d** ZTC, and **e** FG@CuP@ZTC. SEM images of **f** FG@CuP and **g** FG@CuP@ZTC. **h** EDS mapping images of FG@CuP@ZTC. **i** FTIR spectra, **j** XRD patterns, and **k** XPS survey spectra of FG, CuP, ZTC, and FG@CuP@ZTC. **l** High-resolution XPS Cu 2*p*_1/2_, Cu 2*p*_3/2_, and their satellite spectra of CuP and FG@CuP@ZTC. **m** High-resolution XPS P 2*p* spectra of CuP and FG@CuP@ZTC. **n** High-resolution XPS Zn 2*p*_1/2_ and Zn 2*p*_3/2_ spectra of ZTC and FG@CuP@ZTC

The surface morphologies and microstructures of FG, CuP, FG@CuP, ZTC, and FG@CuP@ZTC were characterized by transmission electronic microscopy (TEM) and scanning electron microscope (SEM). As shown in Figs. 2a and S1a, FG presents transparent and wrinkled layered structure. The CuP particle shows cuboid-like structure (Figs. 2b and S1b). From Fig. 2c, the surface of FG is homogeneously covered by amounts of the particles, indicating that the CuP particles are attached on the FG surface successfully. The mechanism of the in situ self-assembling process for FG@CuP is discussed. Firstly, by the strong shear forces during the ball milling process, the NaCMC was adhered to the surface of FG via physical adsorption; thus, the surface was negatively charged [30]. Secondly, the CuP particles were in situ scattered onto the FG surface through the electrostatic interaction [31]. Besides, the diameters of the CuP particles on the FG surface are reduced due to the large aspect ratio FG nanosheets, which restrict the crystallization of CuP [32].

As depicted in Figs. 2d and S1c, ZTC has a polyhedral-like shape. Interestingly, after in situ immobilization, the ZTC layer is uniformly coated onto the FG@CuP surface (Fig. 2e), suggesting the successful preparation of FG@CuP@ZTC. The formation of FG@CuP@ZTC can be ascribed to the strong electrostatic interaction. In addition, after in situ self-assembly, the particle size of ZTC is decreased due to the existence of FG@CuP affecting the crystal growth of ZTC. The SEM images show that FG exhibits the smooth and lamellar-like structure, while its surface becomes extremely rough after the in situ loading of CuP, and the CuP particles are homogeneously dispersed on the FG surface (Fig. 2f). As presented in Fig. 2g, the uniformly distributed ZTC layer on the FG@CuP surface is obviously observed. In addition, the elemental mapping images show that fluorine (F, 10.90 at%), copper (Cu, 4.28 at%), P (3.55 at%), zinc (Zn, 0.63 at%), and N (6.42 at%) are uniformly distributed on the FG@CuP@ZTC surface, indicating that CuP and ZTC are well immobilized on the FG surface (Fig. 2h).

The Fourier transform infrared (FTIR) spectra of FG, CuP, ZTC, and FG@CuP@ZTC are shown in Fig. 2i. For FG, the characteristic peak locates at 1205 cm^−1^ corresponding to the C–F stretching vibration [33]. The FTIR spectrum of CuP exhibits absorption peak at 1436 cm^−1^, which is caused by the stretching vibration of C=C in the benzene ring. Meanwhile, the peak at 1141 cm^−1^ is ascribed to the stretching vibration of P=O bond. The absorption peaks at 1056 and 993 cm^−1^ belong to the stretching vibration of P-O bond and that at 746 cm^−1^ is attributed to P–C bond [34, 35]. For ZTC, the characteristic absorption peaks at 1620, 1512, and 1032 cm^−1^ belong to the stretching vibrations of C=N bond in 3, 5-diamino-1,2,4-triazole, N–H bond, and N–N bond, respectively [36]. The absorption peak around 1557 cm^−1^ is assigned to the bending vibration of C=N [37]. For FG@CuP@ZTC, the characteristic peaks of FG, CuP, and ZTC can be observed in the FTIR spectrum of FG@CuP@ZTC, which verifies the successful in situ loading of CuP and ZTC on the FG surface.

The XRD patterns of FG, CuP, ZTC, and FG@ CuP@ZTC are presented in Fig. 2j. FG exhibits (001) reflection locating in 2*θ*=13.8°, demonstrating its high degree of fluorination and hexagonal features. The (100) reflection peak at around 2*θ*=40.8° corresponds to C–C in plane length of reticular system [38]. The strong diffraction peaks of CuP emerge at 2*θ*=5.7°, 11.5°, and 17.3°, corresponding to the (010), (020), and (030) crystal planes, respectively [39]. For ZTC, the main diffraction signals appear at 2*θ* = 13.0°, 18.6°, 20.5°, 28.5°, and 29.8°, proving its crystalline structure. In the case of FG@CuP@ZTC, all characteristic diffraction peaks of FG, CuP, and ZTC can be detected, suggesting that the in situ immobilization process of CuP and ZTC on the FG surface does not destroy the integrity of their crystal structures.

X-ray photoelectron spectroscopy (XPS) was employed to determine the chemical composition and bonding state of FG, CuP, ZTC, and FG@CuP@ZTC, as displayed in Fig. 2k–n. The peaks of C 1*s*, F 1*s*, and F Auger can be clearly observed in the XPS spectrum of FG. The surface of CuP contains Cu, P, C, and O elements. As exhibited in Fig. 2l, the peaks at 954.18 and 934.18 eV are attributed to Cu 2*p*_1/2_ and Cu 2*p*_3/2_, respectively. The shake-up satellite peak of Cu 2*p*_1/2_ at 962.28 eV and the well-defined doublet satellite peak of Cu 2*p*_3/2_ at 943.58 eV can also be detected. Besides, the peak at 131.98 eV is assigned to P 2*p* (Fig. 2m**)**. The Zn, C, and N elements can be detected on the ZTC surface. The signal at 1045.08 eV is associated with Zn 2*p*_1/2_, and the peak at 1022.08 eV is assigned to Zn 2*p*_3/2_ (Fig. 2n). After in situ loading of CuP and ZTC on the FG surface, the FG@CuP@ZTC contains F, Cu, P, Zn, N, O, and C elements. Furthermore, the Cu 2*p*_1/2_ peak (954.98 eV), satellite peak of Cu 2*p*_1/2_ (963.08 eV), Cu 2*p*_3/2_ peak (934.38 eV), satellite peak of Cu 2*p*_3/2_ (944.28 eV), P 2*p* peak (132.78 eV), Zn 2*p*_1/2_ peak (1045.28 eV), and Zn 2*p*_3/2_ peak (1022.28 eV) of FG@CuP@ZTC appear at higher binding energies than those of CuP and ZTC, indicating the strong interactions between FG, CuP, and ZTC [40].

### Mechanical Performances

**Fig. 3:**
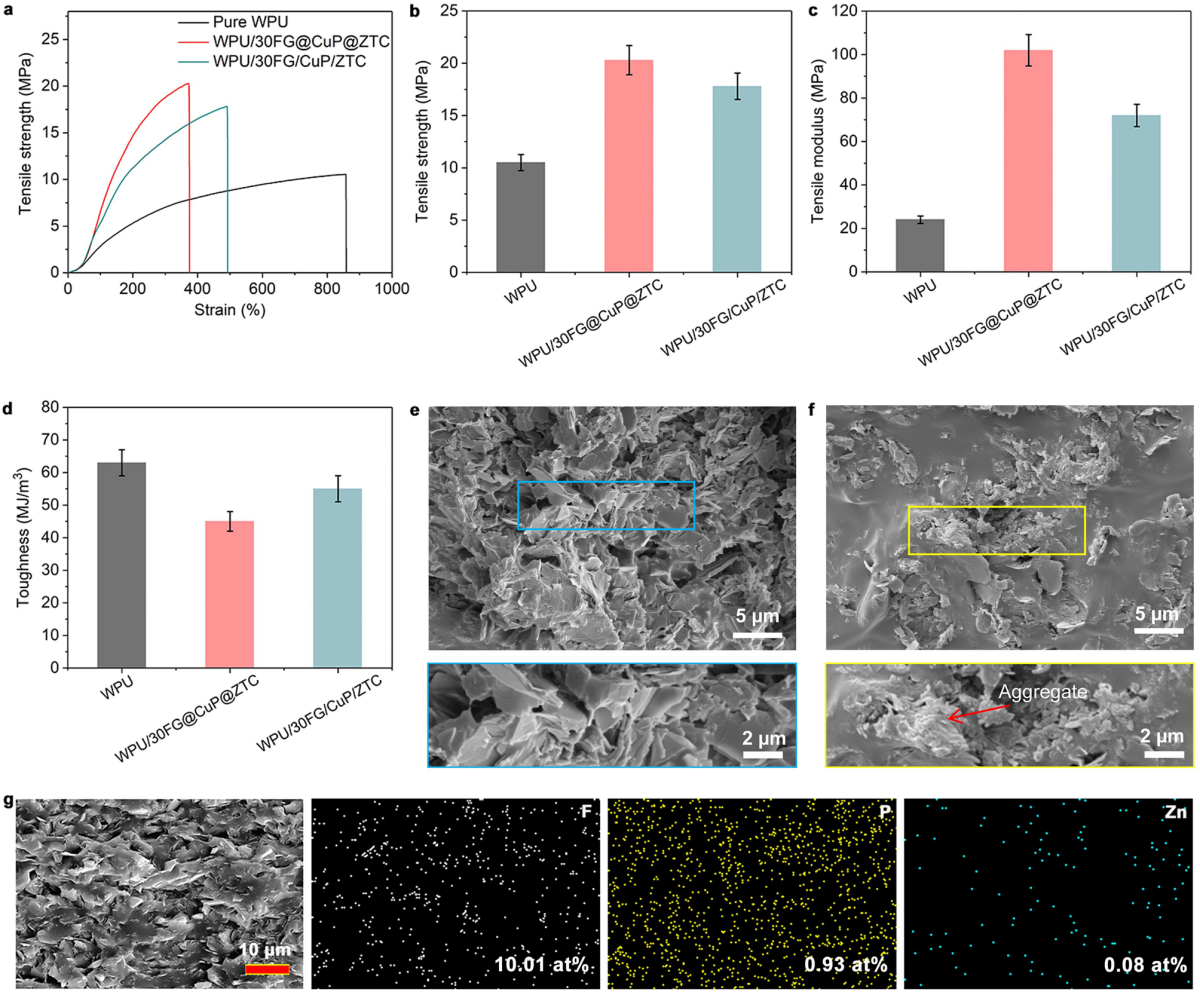
Mechanical performances. **a** Tensile stress–stain curves, **b** tensile strength, **c** tensile modulus, and **d** toughness of pure WPU film and WPU nanocomposite films. SEM images of fracture surfaces for **e** WPU/30FG@CuP@ZTC and **f** WPU/30FG/CuP/ZTC. **g** EDS mappings of the fracture surface of WPU/30FG@CuP@ZTC

In general, ideal microelectronic materials should be mechanically robust for their practical applications. Figure 3a–d, S4, and Table S2 present the mechanical properties of pure WPU film and WPU nanocomposite films. The tensile strength and modulus of pure WPU film are 10.5 and 24 MPa, respectively. With increasing content of FG@CuP@ZTC, the tensile strength and modulus of WPU/FG@CuP@ZTC nanocomposite film gradually increase. When the mass fraction of FG@CuP@ZTC is 30 wt%, the tensile strength and modulus of WPU/30FG@CuP@ZTC nanocomposite film reach the maximum values of 20.3 and 102 MPa, respectively, which are 93.3% and 325% higher than those of pure WPU film. However, the break strains are reduced to different extents. This is because the addition of rigid FG@CuP@ZTC particles results in an increased stiffness or brittleness of the final nanocomposite film. In addition, the immobilization of CuP and ZTC on the FG surface promotes the homogeneous dispersion of FG@CuP@ZTC in the WPU matrix and improves interfacial adhesion between the FG@CuP@ZTC and WPU (as evidenced higher strength and modulus). This can facilitate the interfacial stress transfer from mechanically weak WPU to mechanically strong and stiff FG@CuP@ZTC, which restricts the mutual slippage and the deformation of WPU chains [41]. In comparison with FG@CuP@ZTC, the control WPU/30FG/CuP/ZTC nanocomposite film has smaller tensile strength (17.8 MPa) and elastic modulus (72 MPa) at the same content. This is probably attributed to the poorer dispersion of FG/CuP/ZTC in the WPU matrix, as reflected by the formation of the aggregate (marked by red arrow) which can serve as stress concentration.

To unveil the reinforcing mechanism of FG@CuP@ZTC in WPU, the fracture surfaces of WPU nanocomposite films were imaged by SEM. The WPU film exhibits a relatively smooth fracture surface (Fig. S6), while the WPU/FG@CuP@ZTC nanocomposite films display obvious irregular protuberances. Especially for WPU/30FG@CuP@ZTC (Fig. 3e), the FG@CuP@ZTC is well dispersed throughout the cross section of the WPU matrix without obvious agglomeration, and it is in close contact with each other to form a continuous contact pathway. The FG@CuP@ZTC fillers can be easily identified as they have been pulled out, verifying the strong interfacial interactions between FG@CuP@ZTC and WPU matrix. Due to the in situ growth of CuP and ZTC with the abundant organic P/N-containing segments on the FG surface, the compatibility between FG@CuP@ZTC and WPU is greatly improved, which can promote the entanglement between the FG@CuP@ZTC and WPU molecular chains. As results, the aggregation and sedimentation of FG@CuP@ZTC within the WPU matrix reduces noticeably. Generally, achieving a well-dispersed FG@CuP@ZTC distribution inside the WPU matrix is crucial for the formation of the strong interfacial adhesion and mechanical interlocking between FG@CuP@ZTC and WPU matrix, which is more conducive to stress resistance and can improve the tensile strength [42].

Notably, for the fractured surface of WPU/30FG/CuP/ZTC (Fig. 3f), large agglomerates (a lateral size up to 5 µm) of FG, CuP, and ZTC within the WPU matrix can be detected (marked by yellow square and red arrow). These large agglomerates can serve as defects and stress concentration points, which are not conducive to the effective transfer of the external stress from the relatively soft WPU matrix to the stiff FG, CuP, and ZTC, despite an observed increase in tensile strength as compared to pure WPU [43]. Furthermore, the EDS mapping images of WPU/30FG@CuP@ZTC further illustrate that FG, CuP, and ZTC are well dispersed and embedded in the WPU substrate (Fig. 3g).

### Extraordinary Flame Retardancy

**Fig. 4:**
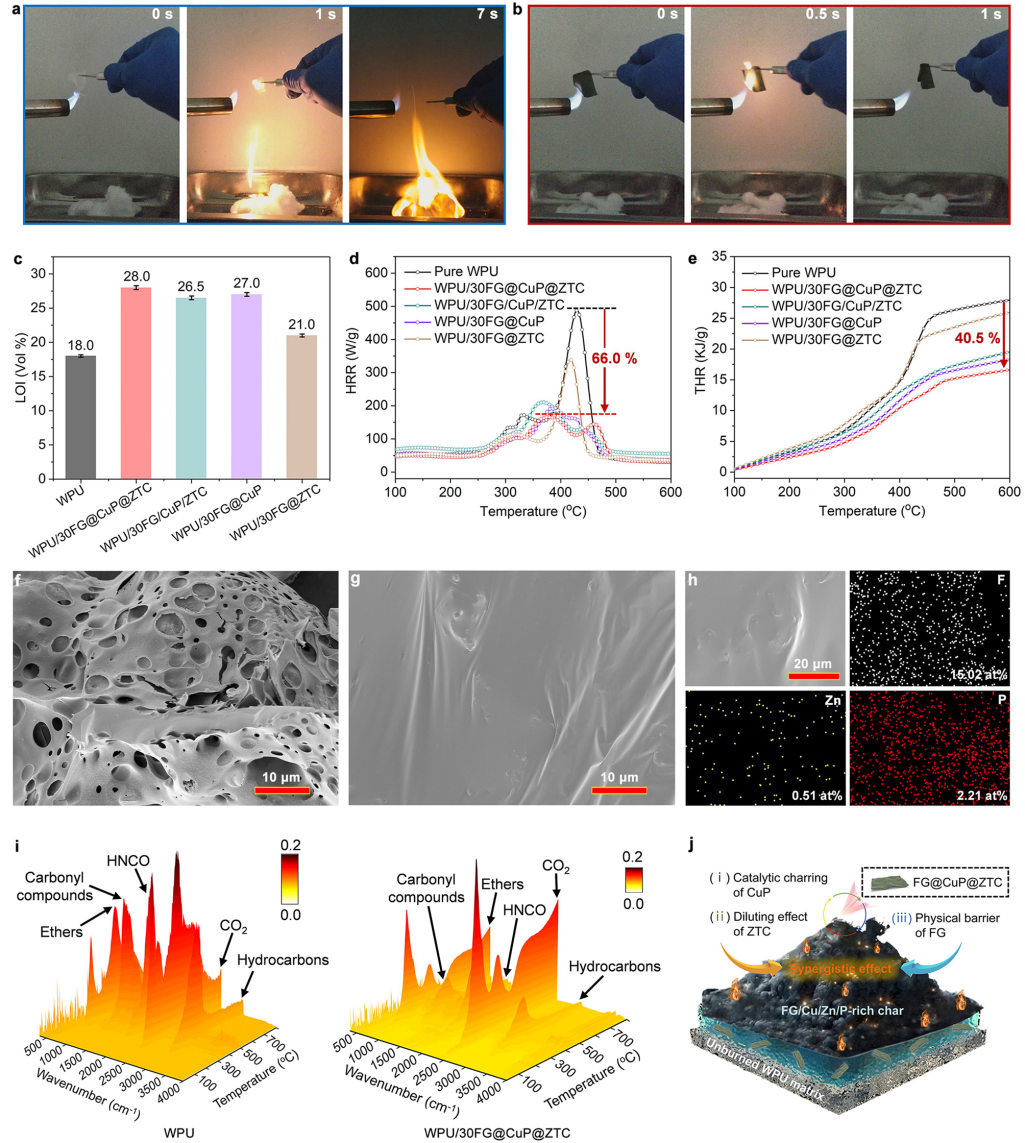
Flame retardancy. Digital photographs of **a** pure WPU film and **b** WPU/30FG@CuP@ZTC nanocomposite film during flame burning test. **c** LOI values, **d** HRR curves, and **e** THR curves for pure WPU film and WPU nanocomposite films. SEM images of char residues for **f** pure WPU and **g** WPU/30FG@CuP@ZTC. **h** EDS mappings of WPU/30FG@CuP@ZTC char. **i** 3D TG-IR spectra of pure WPU and WPU/30FG@CuP@ZTC nanocomposite films. **j** The proposed flame-retardant mechanism of FG@CuP@ZTC for the WPU

The combustion behaviors of pure WPU and WPU nanocomposite films during the flame burning tests were recorded, in which the specimens were ignited by the flame and then moved away. As shown in Fig. 4a, after ignition, the control WPU film begins burning violently, and the droplet phenomenon is serious during the combustion process. The molten dripping quickly ignites the absorbent cotton, and almost no residue is generated after 7 s. In sharp contrast, the WPU/30FG@CuP@ZTC nanocomposite film exhibits a completely different combustion behavior (Fig. 4b). It burns for 0.5 s and subsequently self-extinguishs, and there is no molten dripping generated during the whole combustion process.

Moreover, the LOI value for pure WPU film is only 18.0% (Fig. 4c). As shown in Fig. S9a, upon adding 10 wt% FG@CuP@ZTC, the LOI value increases to 23.5%. When 20 wt% FG@CuP@ZTC is incorporated, the LOI value reaches 26.0%. Under the same loading level of 30 wt%, FG@CuP@ZTC endows WPU film with a higher LOI value (28.0%) than FG@CuP (27.0%), FG@ZTC (21.0%), and simple blending of FG, CuP, and ZTC (26.5%) (Fig. 4c). The above results indicate that the combination of FG, CuP, and ZTC by in situ self-assembly method can obviously improve the fire-retardant performances of WPU film.

Microscale combustion calorimeter (MCC) was carried out to further investigate the combustion behaviors of pure WPU and its nanocomposite films. The heat release rate (HRR) and total heat release (THR) curves are shown in Fig. 4d, e and S9b, c with the data listed in Table S3. The peak heat release rate (PHRR) and THR of pure WPU film are 488 W g^−1^ and 27.9 kJ g^−1^, respectively. Both PHRR and THR values of WPU nanocomposite films containing FG@CuP, FG@ZTC, FG@CuP@ZTC, or FG/CuP/ZTC are decreased compared with those of pure WPU film. In detail, the PHRR and THR values of WPU/10FG@CuP@ZTC are decreased by 41.4% and 12.9%, and those of WPU/20FG@CuP@ZTC are reduced by 52.3% and 24.4% in comparison with those of pure WPU film.

At the same content of 30 wt%, the PHRR values of WPU/30FG@CuP@ZTC, WPU/30FG/CuP/ZTC, WPU/30FG@CuP, and WPU/30FG@ZTC are, respectively, 66.0%, 56.8%, 60.2%, and 30.5% lower than that of pure WPU film. In addition, the THR values of WPU/30FG@CuP@ZTC, WPU/30FG/CuP/ZTC, WPU/30FG@CuP, and WPU/30FG@ZTC are, respectively, decreased by 40.5%, 30.1%, 34.4%, and 7.2% relative to WPU. WPU/30FG@CuP@ZTC presents the lowest PHRR and THR values among all WPU nanocomposite films, further indicating that the flame-retardant efficiency of FG@CuP@ZTC for WPU is higher than those of FG@CuP, FG@ZTC, and FG/CuP/ZTC, which is ascribed to the multiple flame-retardant effects of FG@CuP@ZTC including the catalytic carbonization, gas-phase function, and physical barrier action.

The evaluation of residual char constitutes a significant measure reflecting flame-retardant mechanism in WPU nanocomposite films. The SEM images of the residual char of different WPU samples are shown in Figs. 4f-g and S10. The residual char of pure WPU film displays a loose and porous structure (Fig. 4f), which contributes to the heat and oxygen exchange during combustion. With the FG@CuP@ZTC content rising, the residual char layer becomes rigid and compact (Figs. S10a, b and 4g). As presented in Fig. 4g, adding 30 wt% FG@CuP@ZTC remarkably improves the compactness of char residue, which can protect the WPU matrix from heat and flame. In contrast, the residue chars of WPU/30FG@CuP (Fig. S10c), WPU/30FG@ZTC (Fig. S10d), and WPU/30FG/CuP/ZTC (Fig. S10e) are much looser and less compact compared to that of WPU/30FG@CuP@ZTC, confirming the synergistic effect of FG@CuP@ZTC for WPU film.

Furthermore, the element distribution of the residue char of WPU/30FG@CuP@ZTC was characterized by energy-dispersive X-ray spectroscopy (EDS). As shown in Figs. 4h and S11, the F, Zn, P, and Cu elements are evenly distributed on the surface of WPU/30FG@CuP@ZTC residue char. The Cu, P, Zn, and F-containing compounds can catalyze the formation of the flat, continuous, and rigid residue char, effectively impeding the heat and mass transfer between the gaseous and condensed phases during combustion, thereby leading to the excellent flame-retardant performance [44, 45].

To further understand the modes of action of FG@CuP@ZTC in WPU matrix, its flame inhibition, charring, and barrier-protective layer effects are evaluated quantitatively with the aid of MCC results, as illustrated in the following equations (Eqs.1–3) [46].

1 $${\text{Flame }}\;{\text{inhibition}} = \left( {1 - \frac{{{\text{EHC}}_{c} }}{{{\text{EHC}}_{p} }}} \right) \times 100\%$$

2 $${\text{Charring}}\;{\text{effect}} = \left( {1 - \frac{{{\text{TML}}_{c} }}{{{\text{TML}}_{{p_{c} }} }}} \right) \times 100\%$$

3 $${\text{Barrier - protective}}\;{\text{effect}} = \left( {1 - {{\frac{{{\text{PHRR}}_{c} }}{{{\text{PHRR}}_{p} }}} \mathord{\left/ {\vphantom {{\frac{{{\text{PHRR}}_{c} }}{{{\text{PHRR}}_{p} }}} {\frac{{{\text{THR}}_{c} }}{{{\text{THR}}_{p} }}}}} \right. \kern-0pt} {\frac{{{\text{THR}}_{c} }}{{{\text{THR}}_{p} }}}}} \right) \times 100\%$$

where TML is an abbreviate of total mass loss, and EHC represents effective heat of combustion. At the same loading level of 30 wt%, the as-designed FG@CuP@ZTC has higher flame inhibition (60.3%), charring (21.2%), and barrier-protective layer (42.8%) effects than FG@CuP, FG@ZTC, and FG/CuP/ZTC (see Table S4). For instance, the physical blending of FG, CuP, and ZTC, namely FG/CuP/ZTC, presents a flame inhibition effect of 51.6%, a charring effect of 15.6%, and a barrier-protective layer effect of 38.1%. The aforementioned results indicate that the as-synthesized FG@CuP@ZTC as a flame retardant can impart better retardancy to WPU than the simple blending of FG, CuP, and ZTC due to its improved compatibility with the WPU matrix and the synergistic action of three components.

The evolved gaseous products during the thermal degradation of pure WPU and WPU/30FG@CuP@ZTC nanocomposite films were analyzed by thermogravimetry-infrared (TG-IR) technique. Compared with untreated WPU material, the absorption peak intensities of WPU/30FG@CuP@ZTC in its 3-dimensional (3D) TG-IR spectrum are significantly reduced (Fig. 4i).

The FTIR spectra of pure WPU and WPU/30FG@CuP@ZTC nanocomposite films when their thermal degradation rates reach the maximum are shown in Fig. S12a. Several gaseous decomposition products are remarkably identified by the characteristic absorption peaks. The characteristic peaks of the pyrolysis gaseous products appear at 4000–3500 (H_2_O), 3200–2750 (hydrocarbons), 2360 (CO_2_), 2266 (HCNO), 2190 (CO), 1752 (carbonyl compounds), and 1110 (ethers) cm^−1^ [47]. After incorporating FG@CuP@ZTC into the WPU matrix, the overall absorption peak intensities of these gaseous products are weaker, which reveal that adding FG@CuP@ZTC reduces the release of thermal decomposition products of WPU into the gas phase. From Fig. S12b-f, the characteristic peak intensities of hydrocarbons (2970 cm^−1^), CO_2_ (2360 cm^−1^), HCNO (2266 cm^−1^), carbonyl compounds (1752 cm^−1^), and ethers (1110 cm^−1^) of WPU/30FG@CuP@ZTC are lower than those of pure WPU. During the combustion process of WPU/30FG@CuP@ZTC nanocomposite film, there is lower emission of combustible compounds, which contributes to the formation of more continuous and dense char layers, thus impeding the spread of heat and smoke, thereby improving the fire safety of WPU materials [48].

Based on the abovementioned analyses about both condensed- and gas-phase modes of action, the possible multiple mechanism for the flame retardancy of WPU/FG@CuP@ZTC nanocomposite film is proposed, as depicted in Fig. 4j. During the combustion process, the CuP will undergo thermal decomposition to generate a large number of metaphosphoric and phosphorous acids, which can effectively promote the WPU matrix to dehydrate/carbonize into compact and continuous char layers, reducing the heat and mass transfer between the combustion and non-combustion zones [49, 50]. Simultaneously, FG serves as a physical barrier to inhibit the diffusion of flammable gases into the combustion zone, suppressing the combustion reaction [51]. Meanwhile, ZTC on the FG@CuP surface during combustion can generate intergas (N_2_ and NH_3_), diluting the concentration of combustible gas in the gas phase and removing a large amount of heat [52]. The coexistence of P and N elements fosters a synergistic effect and thus enhances their flame-retardant performances. Besides, the combined actions of Fe-, Zn compounds and FG can make the char layers more robust and rigid, preventing further exchange of heat and mass between the underlying substrate and combustion zone [53, 54]. Consequently, the flame-retardant mechanism of WPU/FG@CuP@ZTC nanocomposite film is a synergistic effect in condensed and gas phases.

### TC and Dielectric Performances

**Fig. 5:**
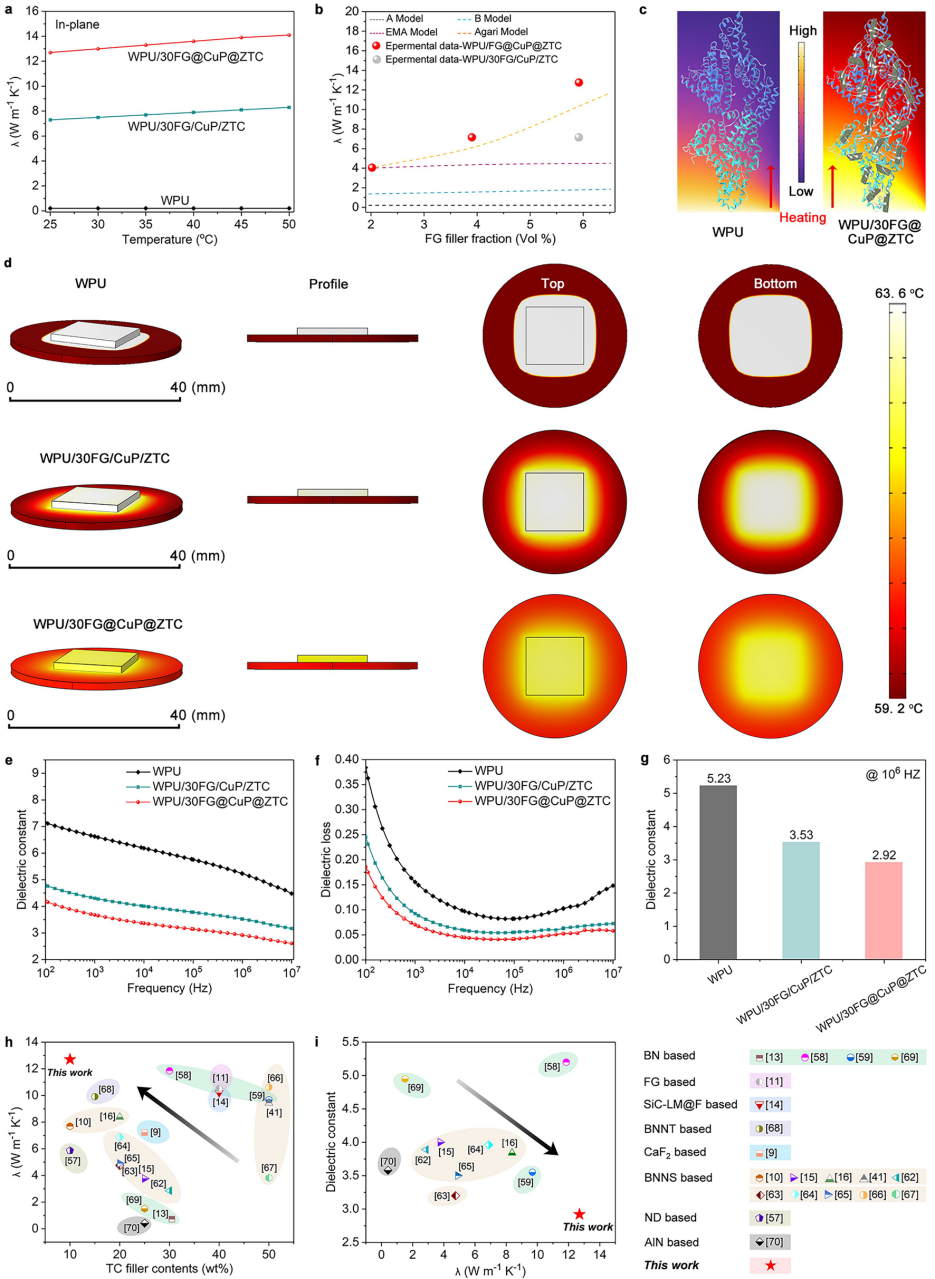
TC and dielectric performances.** a** In-plane *λ* of WPU samples as a function of temperature. **b** *λ* as a function of the volume fraction of FG together with theoretical prediction values by Eqs. 4–7. **c** Schematic diagram of TC mechanism for WPU nanocomposite films. **d** Modeling and calculation of the temperature for WPU, WPU/30FG/CuP/ZTC, and WPU/30FG@CuP@ZTC. **e** *ε* and **f** dielectric loss curves of WPU samples. **g** *ε* of WPU samples at 10^6^ Hz. **h** Comparisons of the variation in *λ* of TC materials for different systems. **i** Comparisons of *λ* and *ε* obtained in this work and previously reported TC materials

Figures 5a and S13 show the in-plane *λ* of WPU samples at different temperatures. The *λ* of WPU nanocomposite films remains stable between 25 and 50 °C, which facilitates long-term applications. The *λ* of WPU/FG@CuP@ZTC nanocomposite films increases with increasing content of FG@CuP@ZTC, mainly due to the gradually increasing probability of forming TC pathways in the WPU matrix. Strikingly, at the same loading level, the *λ* of WPU/30FG@CuP@ZTC is higher than that of WPU/30FG/CuP/ZTC. This can be attributed to the in situ immobilization of CuP and ZTC on the FG surface, which induces strong interactions between FG@CuP@ZTC and WPU matrix, thereby reducing the interfacial thermal resistance [2].

The *λ* of WPU/10FG@CuP@ZTC and WPU/20FG@CuP@ZTC at 25 °C is 4.0 and 7.1 W m^−1^ K^−1^, respectively, exhibiting 1900% and 3450% higher than that of pure WPU film (0.2 W m^−1^ K^−1^). Moreover, WPU/30FG/CuP/ZTC presents a *λ* of 7.3 W m^−1^ K^−1^, while that of WPU/30FG@CuP@ZTC increases to 12.7 W m^−1^ K^−1^ by 74%. The FG/CuP/ZTC fillers are poorly dispersed in the WPU matrix and form obvious aggregations, which cannot form efficient thermal conduction pathways. Therefore, the heat transfer is slow, and the heat flow is scattered and disordered. As a result, the TC performance improvement of WPU/30FG/CuP/ZTC is not satisfying.

Figure 5b presents the fitting results of *λ* of WPU/FG@CuP@ZTC nanocomposite film through the following models (Eqs. 4–7) to clarify the effects of the heterostructured FG@CuP@ZTC on the TC performance of WPU nanocomposite film [30, 55, 56].

4 $$\lambda_{c}^{{A\;{\text{Model}}}} = 1/[\left( {1 - V_{f} } \right)/\lambda_{m} + V_{f} /\lambda_{f}$$

5 $$\lambda_{c}^{{B\;{\text{Model}}}} = \lambda_{m}^{{\left( {1 - V_{f} } \right)}} + \lambda_{f}^{{V_{f} }}$$

6 $$\lambda_{c}^{{ EMA\;{\text{Model}}}} = \lambda_{f} \left[ {\frac{{3\lambda_{m} + 2V_{f} \left( {\lambda_{f} - \lambda_{m} } \right)}}{{\left( {3 - V_{f} } \right)\lambda_{f} + \lambda_{m} + \frac{{R\lambda_{m} \lambda_{f} V_{f} }}{H}}}} \right]$$

7 $$\log \lambda_{c}^{{Agari\;{\text{Model}}}} = V_{f} C_{2} \log \lambda_{f} + (1 - V_{f} )\log \left( {C_{1} \lambda_{m} } \right)$$

Among them, *V*_f_ is the volume fraction of the FG fillers; *λ*_c_, *λ*_m_, and *λ*_f_ are the *λ* values of WPU/FG@CuP@ZTC nanocomposite films, WPU matrix, and FG, respectively. H is the thickness of FG and R is the thermal resistance. C_1_ is a factor relating to the crystallinity and crystal size, and C_2_ is a factor relating the ease in forming TC networks. From Fig. 5b, the tested *λ* values in this work are in agreement with the predicted ones by Agari model to some extent. Figures 5c and S14 illustrate the proposed TC mechanism of the as-prepared WPU/FG@CuP@ZTC nanocomposite films. For WPU/FG@CuP@ZTC nanocomposite films, the CuP and ZTC on the FG surface can avoid the aggregation of FG@CuP@ZTC and improve the interfacial interactions between FG@CuP@ZTC and WPU matrix, thus depressing the interfacial thermal resistances, reducing the phonon scattering probability, and strengthening phonon propagation [57, 58]. Consequently, the efficient formations of numerous TC pathways are realized and the heat transfers are increased, and the *λ* of WPU/FG@CuP@ZTC is greatly enhanced [59].

Furthermore, the heat dissipation and heat flow during heating process in WPU, WPU/30FG/CuP/ZTC, and WPU@30FG@CuP@ZTC are simulated through finite element simulation (FES). In the current case, the transient-state finite element methodology is applied to simulate the transient thermal response and temperature distributions. The circular samples (diameter of 40 mm) with different thermal conductivities are contacted with the bottom surface of square heat source with constant internal heat generation. The thickness of all the samples is 100 μm. As shown in Fig. 5d, the heat transfers faster from the heat source to boundary in WPU@30FG@CuP@ZTC than that in pure WPU and WPU/30FG/CuP/ZTC, which can be ascribed to the superior *λ* of WPU@30FG@CuP@ZTC. In addition, the heat source with WPU@30FG@CuP@ZTC exhibits the lowest center temperature, demonstrating the highest heat transfer efficiency of WPU@30FG@CuP@ZTC. Overall, WPU@30FG@CuP@ZTC presents excellent heat dissipation and efficient thermal management capabilities, which can be as a promising candidate for real thermal management such as microelectronic cooling application.

Figures 5e, f and S15a,b present the *ε* and dielectric loss of pure WPU and WPU nanocomposite films at 10^2^–10^7^ Hz and 25 °C. The *ε* and dielectric loss of WPU/FG@CuP@ZTC decrease gradually as the FG@CuP@ZTC content increases. Notably, at the loading level of 30 wt%, the *ε* and dielectric loss of WPU/30FG/CuP/ZTC nanocomposite film are higher than those of WPU/30FG@CuP@ZTC nanocomposite film, which is ascribed to the interfacial polarization between FG/CuP/ZTC and WPU matrix [17, 60, 61]. The *ε* of WPU samples at 10^6^ Hz is shown in Figs. 5g and S15c. The *ε* of WPU/10FG@CuP@ZTC and WPU/20FG@CuP@ZTC reaches 4.53 and 3.90, respectively, which are 13.4% and 25.4% lower than that of pure WPU. The WPU/30FG@CuP@ZTC nanocomposite film exhibits the lowest *ε* of 2.92, which is reduced by 17.3% relative to that of WPU/30FG/CuP/ZTC nanocomposite film, confirming its low dielectric performance.

The comparisons of the variation in in-plane *λ* of TC materials at 25 °C for different systems are shown in Fig. 5h and Table 1. Notably, the WPU/30FG@CuP@ZTC nanocomposite film exhibits the highest *λ* at the lowest TC filler content of 10 wt%, far surpassing those of TC nanocomposites-based BNNS [10, 15, 16, 41, 62–67], FG [11], boron nitride nanotubes (BNNT) [68], calcium fluoride (CaF_2_) [9], fluorine-terminated functionalized liquid metal/silicon carbide (SiC-LM@F) [14], nanodiamond (ND) [57], boron nitride (BN) [13, 58, 59, 69], and aluminum nitride (AlN) [70], which demonstrates the preparation methodological advantages of WPU/30FG@CuP@ZTC nanocomposite film.

**Table 1:** Comparison of TC properties and *ε* of WPU/FG@CuP@ZTC and previously reported TC materials

Sample	TC filler	TC filler type	TC filler content (wt%)	In-plane *λ* (W m^−1^ K^−1^)	*ε* at 10^6^ Hz	References
PVDF/hBN/MWCNTs-SiO_2_	hBN/MWCNTs-SiO_2_	BN based	25	1.51	4.95	2020 [69]
PI/PDA-BNF@BNNP	PDA-BNF@BNNP	BN based	30	11.85	5.2	2022 [58]
PTFE /hBN-KH550	hBN-KH550	BN based	30.5	0.722	–	2017 [13]
PNF/m-BN	m-BN	BN based	50	9.68	3.55	2024 [59]
ANF/f-G	f-G	FG based	40	10.52	–	2021 [11]
PVA/SiC-LM@F	SiC-LM@F	SiC-LM@F based	40	10.2	–	2022 [14]
ANF/PEI/BNNT	BNNT	BNNT based	15	9.91	–	2021 [68]
PI/IL-CaF_2_	IL-CaF_2_	CaF_2_ based	25	7.22	–	2024 [9]
PAI/BNNS/BNQD	BNNS/BNQD	BNNS based	10	7.69	–	2019 [10]
PLA/BNNS	BNNS	BNNS based	20	4.9	~ 3.5	2024 [65]
PI/MBN-AgNW	MBN-AgNW	BNNS based	20	8.38	3.84	2024 [16]
PEI /BNNSs@MWCNTs	BNNS@MWCNTs	BNNS based	20	6.88	3.96	2022 [64]
PI/BNNS-AgNW	BNNS-AgNW	BNNS based	20	4.75	3.2	2020 [63]
PDMS/BNNS@Ag	BNNS@Ag	BNNS based	25	3.77	4.0	2023 [15]
RTV-2SR /BNNS@Al_2_O_3_	BNNS@Al_2_O_3_	BNNS based	30	2.86	3.89	2021 [62]
ANF/AgNWs@BNNS	AgNWs@BNNS	BNNS based	50	9.44	–	2023 [41]
PANF/APS-BNNS	APS-BNNS	BNNS based	50	10.62	–	2024 [66]
PDMS/BNNS	BNNS	BNNS based	50	3.82	–	2024 [67]
PVA/ND@PDA	ND@PDA	ND based	10	5.864	–	2020 [57]
PS/AlN	AlN	AlN based	25	0.418	3.58	2016 [70]
WPU/FG@CuP@ZTC	FG	FG based	10	12.7	2.92	This work

As illustrated in Fig. 5i and Table 1, at room temperature, the *λ* value and the *ε* of WPU/30FG@CuP@ZTC nanocomposite film obtained in this work are at higher levels compared to those of previously reported materials. Clearly, compared to the nanocomposites with dopamine-modified hexagonal boron nitride flakes and nanoparticles (PDA-BNF@BNNP) [58], m-BN [59], hexagonal boron nitride/silica-coated carbon nanotubes (hBN/MWCNTs-SiO_2_) [69], silver nanoparticle (AgNP)-decorated with boron nitride nanosheets (BNNS@Ag) [15], modified boron nitride nanosheet/silver nanowires (MBN-AgNW) [16], boron nitride nanosheets/alumina (BNNS/Al_2_O_3_) [62], boron nitride nanosheets/silver nanowires (BNNS-AgNW) [63], BNNS/MWCNTs [64], BNNS [65], and AlN [70], our WPU nanocomposite film shows the highest *λ* and the lowest *ε* at 10^6^ Hz, satisfying the requirements of advanced microelectronic devices.

**Fig. 6:**
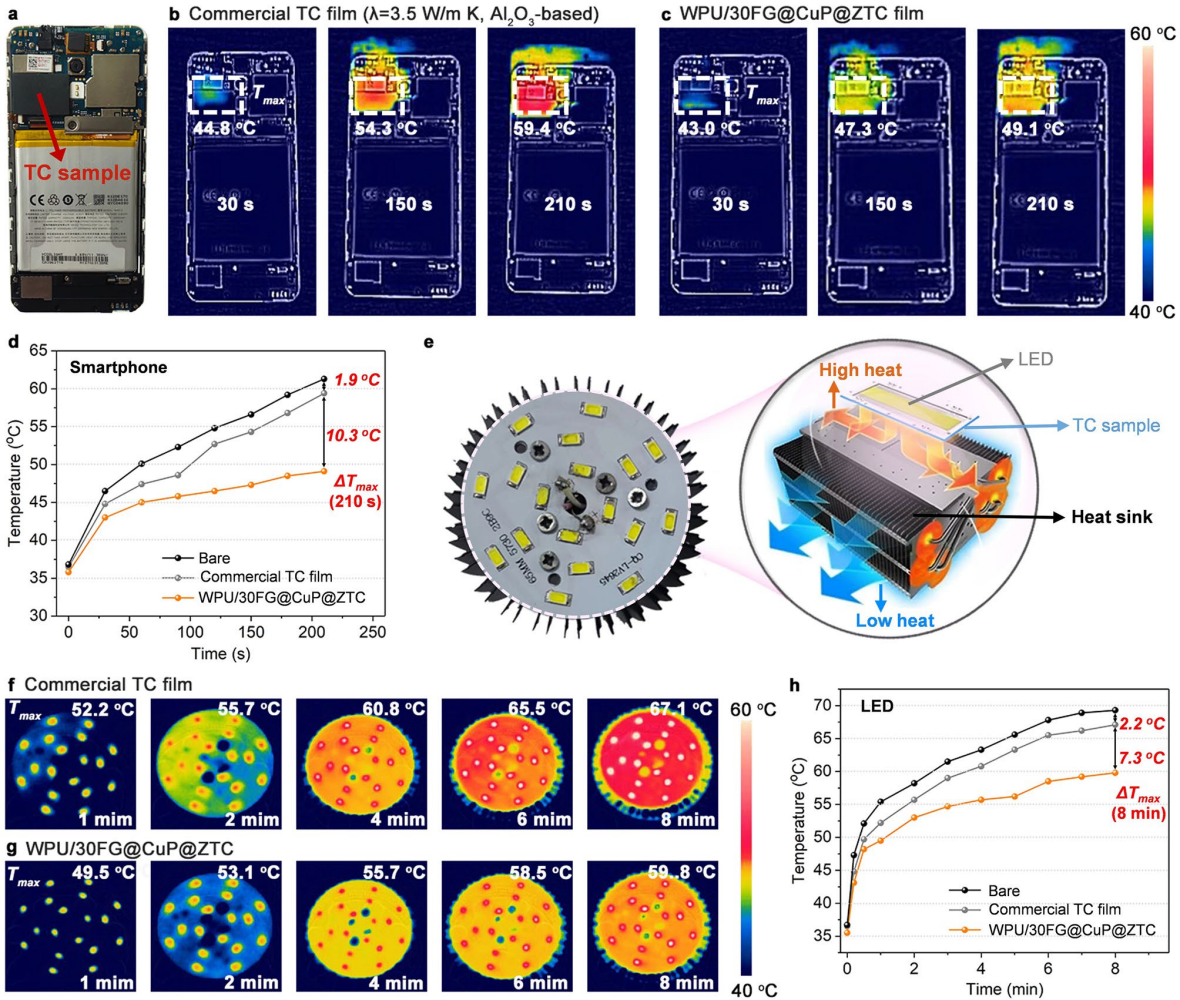
Thermal management demonstrations of WPU/30FG@CuP@ZTC nanocomposite film.** a** Optical photograph of the CPU of smartphone integrated with WPU/30FG@CuP@ZTC nanocomposite film. The infrared thermal images of the control smartphone under different working conditions when using **b** commercial film and **c** WPU/30FG@CuP@ZTC nanocomposite film. **d** The corresponding surface temperature variations. **e** Schematic of the TC samples integrated between the LED chip and heat sink. The maximum temperature evolution of the LED chip and the infrared thermal images of LED chip with **f** commercial film and **g** WPU/30FG@CuP@ZTC nanocomposite film, and **h** the corresponding temperature–time relationship curves

To investigate the heat dissipation capability of WPU/30FG@CuP@ZTC nanocomposite film as TC material in practical applications, it is used to reduce the operating temperature of central processing unit (CPU) of smartphone. The thickness of WPU/30FG@CuP@ZTC nanocomposite film and commercial film is 100 μm. As schematically presented in Fig. 6a, the WPU/30FG@CuP@ZTC sample is packaged on the surface of CPU. As shown in Fig. 6b-d, compared with commercial film, the CPU with WPU/30FG@CuP@ZTC nanocomposite film exhibits more uniform heat distribution and lower surface temperature during the whole operation process. When working for 210 s, the maximum temperature (*T*_max_) values of CPU integrated with commercial film and WPU/30FG@CuP@ZTC film are around 59.4 and 49.1 °C, respectively. The maximum temperature difference (Δ*T*_max_) between commercial film and WPU/30FG@CuP@ZTC film is as high as 10.3 °C, while the Δ*T*_max_ of the CPU with commercial film and the bare CPU is only 1.9 °C. Obviously, the WPU/30FG@CuP@ZTC nanocomposite film with a high *λ* reduces the heat aggregation and enhances the heat dissipation as a non-combustible thermal spreader.

To further demonstrate the heat dissipation performance of the WPU/30FG@CuP@ZTC nanocomposite film in real electronics, it is used as a heat spreader for cooling light-emitting diode (LED). As presented in Fig. 6e, the WPU/30FG@CuP@ZTC nanocomposite film is placed between the aluminum heat sink and the LED chip. The LED chip and WPU/30FG@CuP@ZTC nanocomposite film are fixed to the aluminum heat sink with four screws to reduce the gaps between the LED chip and WPU/30FG@CuP@ZTC nanocomposite film. The *T*_max_ of the LED chip as a function of running time was recorded by an infrared thermal imager and the resulting temperature profiles as well as the corresponding images displayed in Fig. 6f–h. Notably, the LED chip integrated with WPU/30FG@CuP@ZTC nanocomposite film demonstrates lower-temperature rise compared to that with the commercial film. At 8 min, the *T*_max_ of the LED chip with WPU/30FG@CuP@ZTC reaches 59.8 °C, which is 7.3 °C lower than that of the LED chip with commercial film (67.1 °C). In addition, the *T*_max_ of the LED chip with commercial film can only be reduced by about 2.2 °C, in comparison with the bare LED chip. Obviously, the WPU/30FG@CuP@ZTC nanocomposite film shows more efficient heat transfer capability than commercial film, which is expected to be used as a high-performance alternative in microelectronic equipments.

## Conclusions

In summary, we have engineered a multifunctional heterostructured nanoadditive, FG@CuP@ZTC, which was then used to prepare WPU/FG@CuP@ZTC nanocomposite films by bionic LBL blade coating. The as-prepared WPU nanocomposite film achieved high tensile strength, satisfactory flame retardancy, outstanding TC performances, and low *ε*. The combination of a satisfactory flame retardancy (LOI value increased by 55.6%, and PHRR and THR decreased by 66.0% and 40.5%, respectively), superior reinforcing effect (tensile strength increased by 93.3%), a high *λ* of 12.7 W m^−1^ K^−1^, and a low *ε* of 2.92 was achieved in the WPU/30FG@CuP@ZTC nanocomposite film due to the synergistic action of FG, CuP, and ZTC. The WPU nanocomposite film has demonstrated the strong heat dissipation capability and superior cooling efficiency in high powder smartphone and LED modules. Consequently, this work provides an innovative strategy for the fabrication of multifunctional polymer nanocomposite film with outstanding flame retardancy, high *λ*, and low *ε*, and this high-performance film holds a promising potential application in microelectronic devices and their components.

## Supplementary Information

Below is the link to the electronic supplementary material.

Supplementary file1
