# Supplementary material for: An Engineered Heterostructured Trinity Enables Fire-Safe, Thermally Conductive Polymer Nanocomposite Films with Low Dielectric Loss
Source: Nanomicro Lett. 2025 Feb 26;17:168. doi: 10.1007/s40820-025-01681-9 (PMC11865423; doi:10.1007/s40820-025-01681-9)
Supplement: Supplementary file 1 — Supplementary file1 [file 40820_2025_1681_MOESM1_ESM.docx]

Supporting Information for

**An Engineered Heterostructured Trinity Enables Fire-Safe, Thermally Conductive Polymer Nanocomposite Films with Low Dielectric Loss**

Qiang Chen^1^, Jiabing Feng^3^, Yijiao Xue^4^, Siqi Huo^5^, Toan Dinh^5^, Hang Xu^1,2,^ *, Yongqian Shi^6^, Jiefeng Gao^7^, Long-Cheng Tang^8^, Guobo Huang^9,^ *, Weiwei Lei^10^, Pingan Song^11,^ *

^1^Key Laboratory of Integrated Regulation and Resource Development on Shallow Lakes, Ministry of Education, College of Environment, Hohai University, No.1 Xikang Road, Nanjing 210098, P. R. China

^2^Suzhou Research Institute of Hohai University, Suzhou 215000, P. R. China

^3^College of Biological, Chemical Sciences and Engineering, Jiaxing University, Jiaxing 314001, P. R. China

^4^Institute of Chemical Industry of Forest Products, Chinese Academy of Forestry (CAF), Nanjing 210042, P. R. China

^5^Centre for Future Materials, School of Engineering, University of Southern Queensland, Springfield, 4300 Australia

^6^ College of Environment and Safety Engineering, Fuzhou University, 2 Xueyuan Road, Fuzhou 350116, P. R. China

^7^School of Chemistry and Chemical Engineering, Yangzhou University, Yangzhou 225002, P. R. China

^8^Key Laboratory of Organosilicon Chemistry and Material Technology of MoE, College of Material, Chemistry and Chemical Engineering, Hangzhou Normal University, Hangzhou 311121, P. R. China

^9^School of Pharmaceutical and Chemical Engineering, Taizhou University, Taizhou 318000, P. R. China

^10^School of Science, RMIT University, Melbourne, VIC, 3000, Australia

^11^Centre for Future Materials, University of Southern Queensland, Springfield, 4300 Australia

*Corresponding authors. E-mail: [pingansong@gmail.com](mailto:pingansong@gmail.com) and [pingan.song@usq.edu.au](mailto:pingan.song@usq.edu.au) (Pingan Song); [xuhang810826@163.com](mailto:xuhang810826@163.com) (Hang Xu ); [huangguobo@tzc.edu.cn](mailto:huangguobo@tzc.edu.cn) (Guobo Huang)

**S1 Supplementary Experimental Section**

**S1.1 Materials Characterizations**

Transmission electron microscopy (TEM) measurements were carried out on a JEOL JEM-2100F equipment (Nippon Electric Motor, Japan). Scanning electron microscope (SEM, Hitachi S-2360N, Japan) with an attachment of Energy Dispersive spectrometer (EDS) was used to observe morphologies, and the samples were sprayed with gold before testing. Fourier transform infrared (FTIR) spectra of the samples were captured on QUINOX 55 equipment (Bruker, Germany) to characterize the chemical groups composition. X-ray diffraction (XRD) analysis was performed using a Rigaku diffractometer with Cu Kα radiation at 40 kV. X-ray photoelectron spectroscopy (XPS) spectra of the samples were tested by the thermo Scientific K-Alpha (USA). A laser particle size analyzer (Mastersizer 3000, Malvern Panalytical, England) was used to measure the size distribution of FG and FG@CuP@ZTC.

The limited oxygen index (LOI) measurement was conducted using the LOI instrument (HC-2, China) according to according to GB/T 2406-2009 standard. The heat release rate was observed by a microscale combustion calorimeter (MCC, GOVMARK MCC-2, USA) to assess the flame resistance. 3-5 mg of samples were added into sample cell and heated with a rate of 1 °C s^-1^ under the protection of a carried gas which was made up of nitrogen (80 mL min^-1^) and oxygen (20 mL min^-1^). Thermogravimetric-Fourier transform infrared spectroscopy (TG-IR) was performed on a thermal gravimeter (NETZSCH 209F3, Germany) and fourier transform infrared spectrometer (Bruker TENSOR27, Germany) from 30 to 700 °C with a heating rate of 20 °C/min in N_2_, and the sample weight was in the range of 5-10 mg.

The tensile strengths were tested on an AG-X plus universal tensile machine (Shimadzu Instruments, Japan) with a rectangle sample size of 200 × 100 mm and loading rate of 13.3 mm/min at room temperature, and the final tensile strengths were averaged from at least five-times results. For the resilience measurement, the samples are subjected to a cyclic strain of 50% for 5 times on an electromechanical universal testing machine (E44.104, MTS, China).

The thermal diffusivity values (α, mm^2^/s) of the samples were obtained by a “laser flash” technique (LFA-467, NETZSCH, Germany). The specific heat capacity (C_p_, J g^-1^ K^-1^) was measured using a differential scanning calorimetry (DSC214, NETZSCH, Germany), and the density (ρ, g cm^-3^) was measured by a density balance following the Archimedes principle with ethanol as the medium. The thermally conductivity (λ, W m^-1^ K^-1^) was calculated according to the following equation: λ = α × C_p_ × ρ, and three specimens of each material were measured. Surface temperature responses were measured with an infrared thermograph (FLIR, E5, USA).

The dielectric properties (dielectric constant and dielectric loss) of samples were tested by a Broadband Dielectric Spectroscopy (Novocontrol Concept 80, Germany) at room temperature over a wide frequency range from 10^2^ to 10^7^ Hz.

**S1.2 Synthesis of FG@ZTC**

Briefly, FG, 0.1 g of NaCMC and 300 mL H_2_O were added to a ball milling tank containing zirconium beads. After 12 h of ball milling at a rotation speed of 1000 rpm, the FG aqueous dispersion was obtained. Afterward, 3,5-diamino-1,2,4-triazole was placed into 100 ml H_2_O with ultrasonication for 0.5 h, followed by the addition of the FG aqueous dispersion and stirred for 0.5 h. After that, ZnSO_4_·7H_2_O aqueous solution was added dropwise into above FG dispersion with 3,5-diamino-1,2,4-triazole, and reacted for another 8 h under. Finally, the target FG@ZTC with a weight ratio of FG and ZTC *ca.* 1: 2 was collected by centrifugation from the slurry, washing with the H_2_O, and freeze-drying (pressure: 10 Pa and temperature: -80 °C) for 48 h.

**S2 Supplementary Figures and Tables**


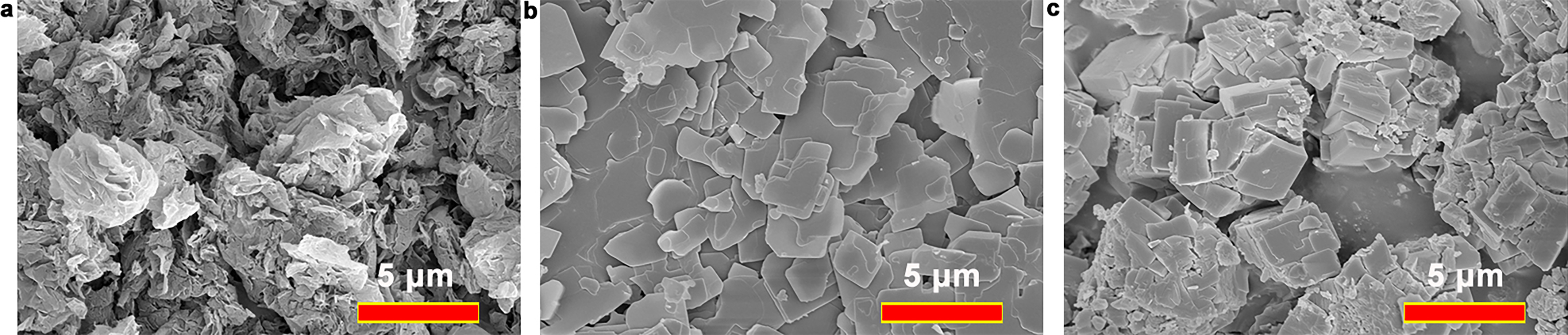


**Fig. S1** The SEM image of **a** FG, **b** CuP, and **c** ZTC


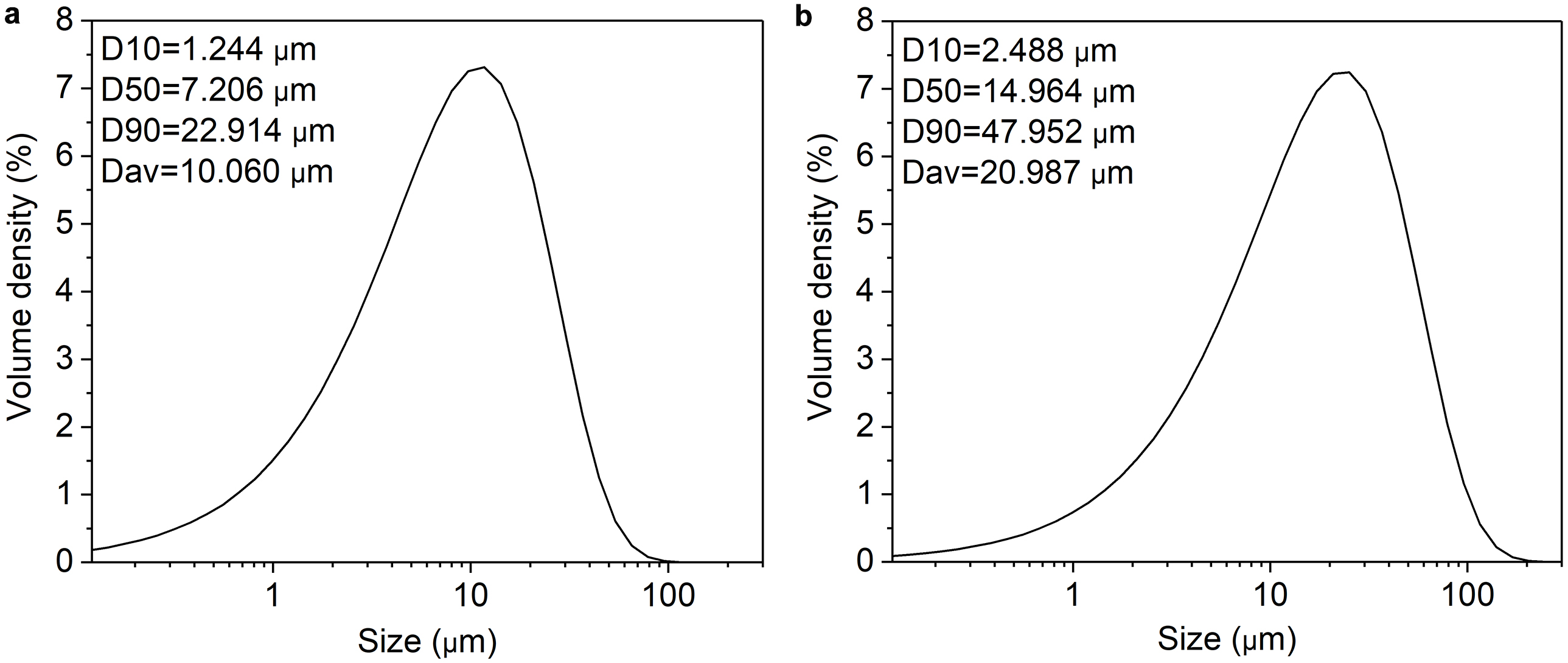


**Fig. S2** The size distributions of **a** FG and **b** FG@CuP@ZTC

Here, D refers to the volume median diameter, and it is derived from the laser diffraction results to reflect the cumulative distribution of the particle size. The number indicates how many percentages of the particles are smaller than this value (e.g., Dv50 means half of the particles are smaller than a certain size). And Dav is the De Brouckere mean diameter, which is the volume moment mean diameter.


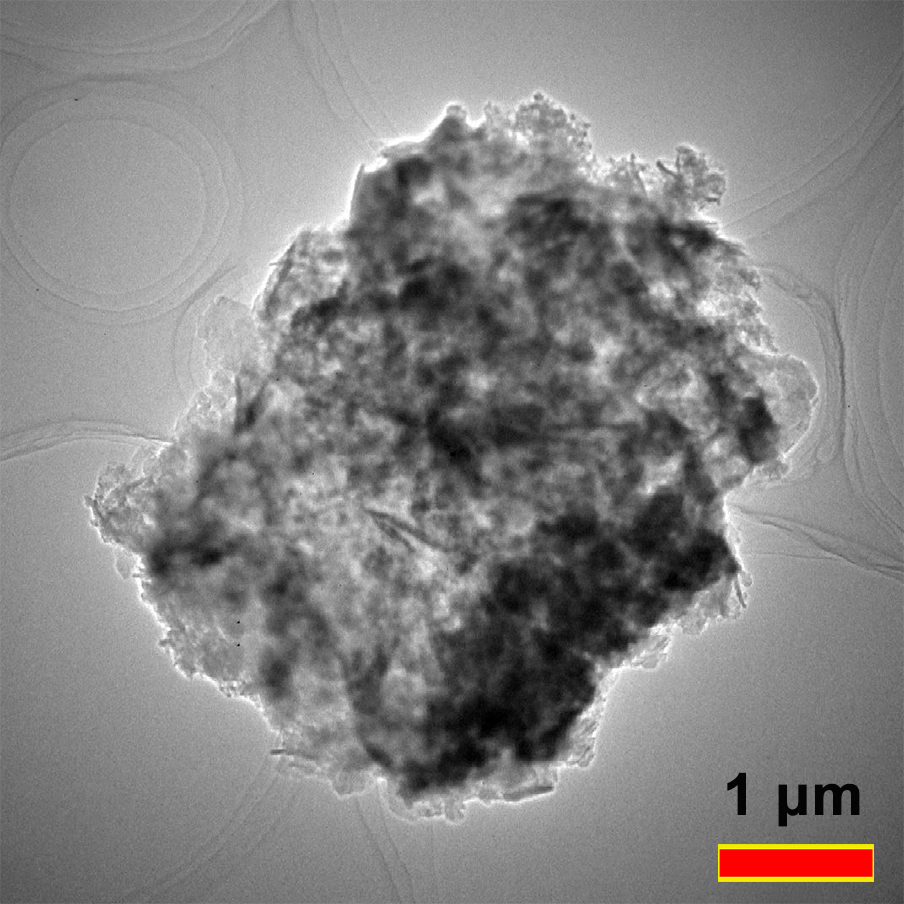


**Fig. S3** TEM image of FG@CuP after the secondary ball milling


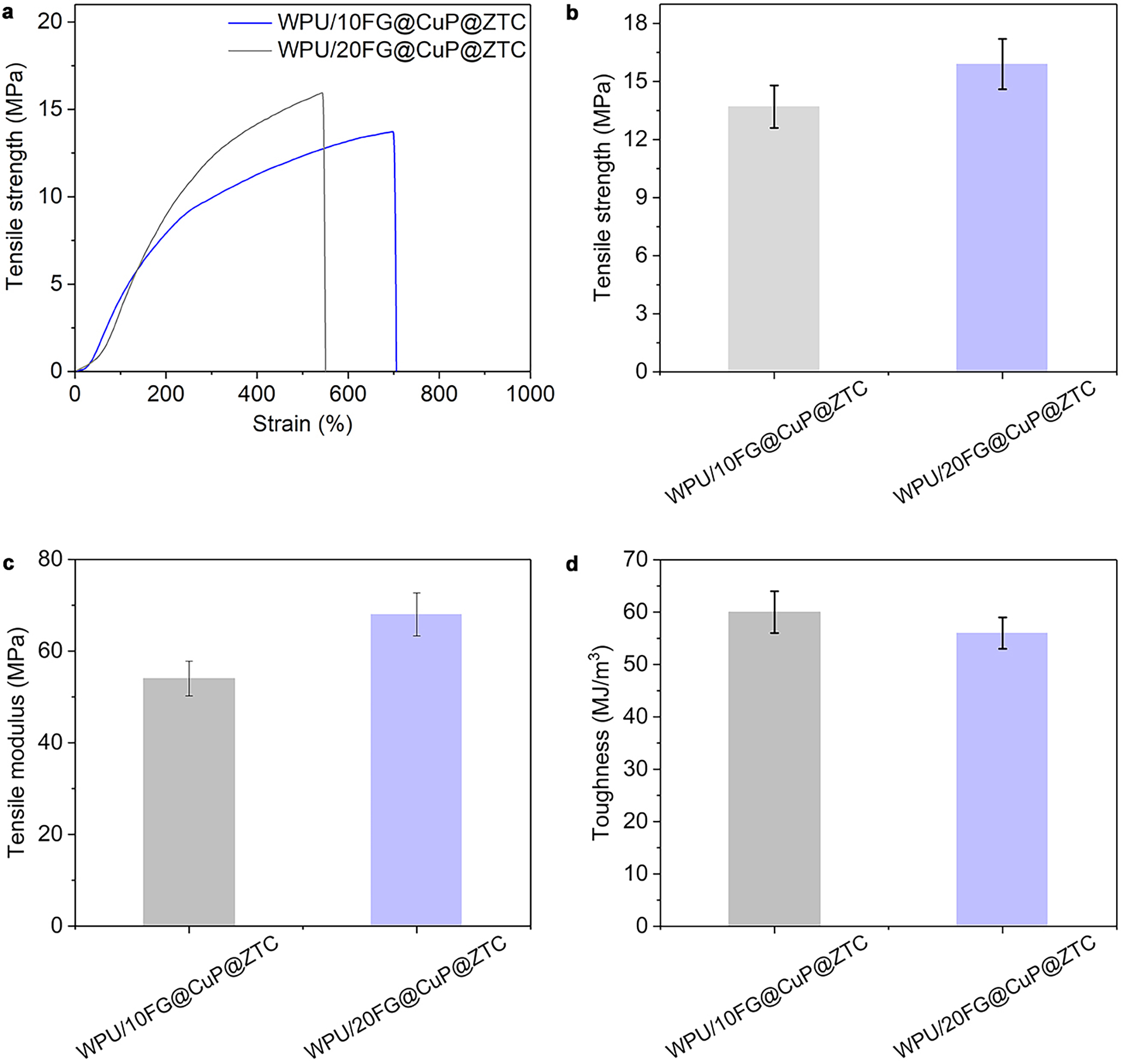


**Fig. S4 a** Tensile stress-stain curves, **b** tensile strength, **c** tensile modulus, and **d** toughness of different WPU samples


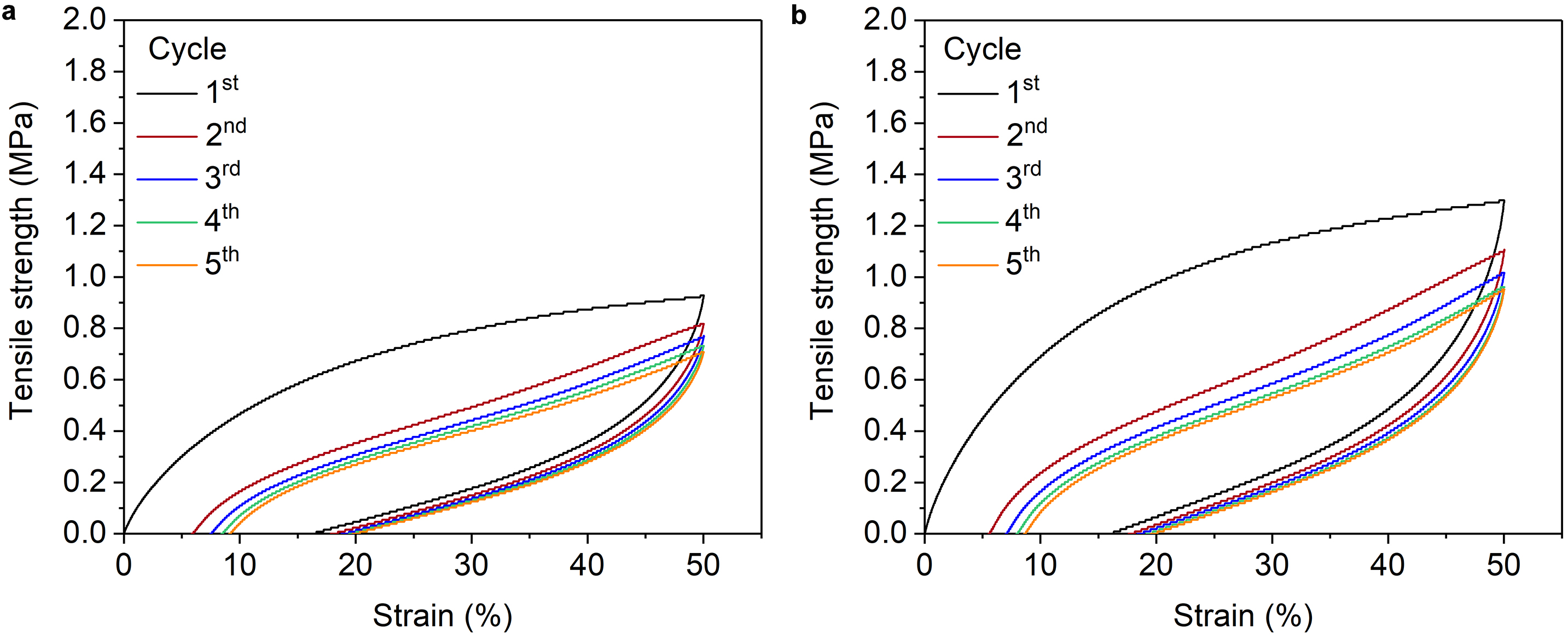


**Fig. S5** The cyclic loading-unloading curves of **a** pure WPU film and **b** WPU/30FG@CuP@ZTC nanocomposite film


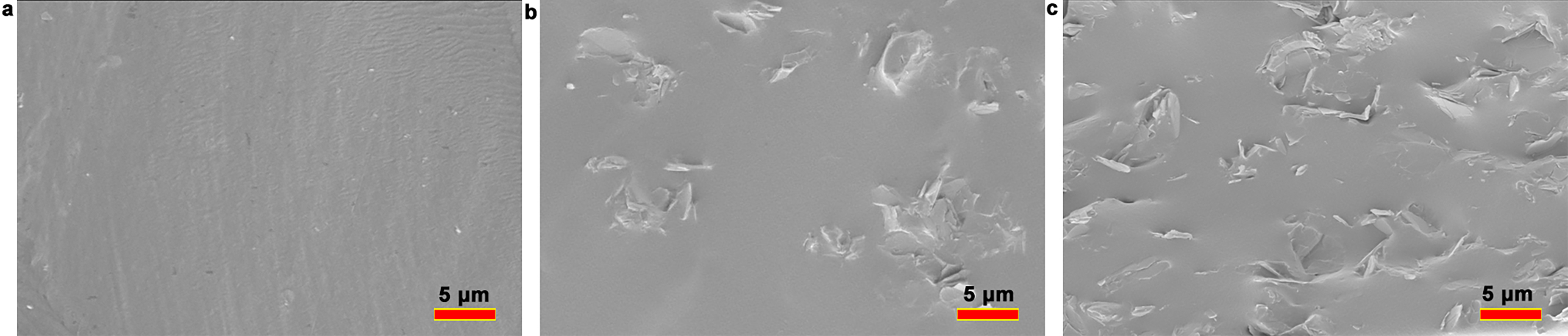


**Fig. S6** SEM images of fracture surfaces for **a** pure WPU, **b** WPU/10FG@CuP@ZTC, and **c** WPU/20FG@CuP@ZTC


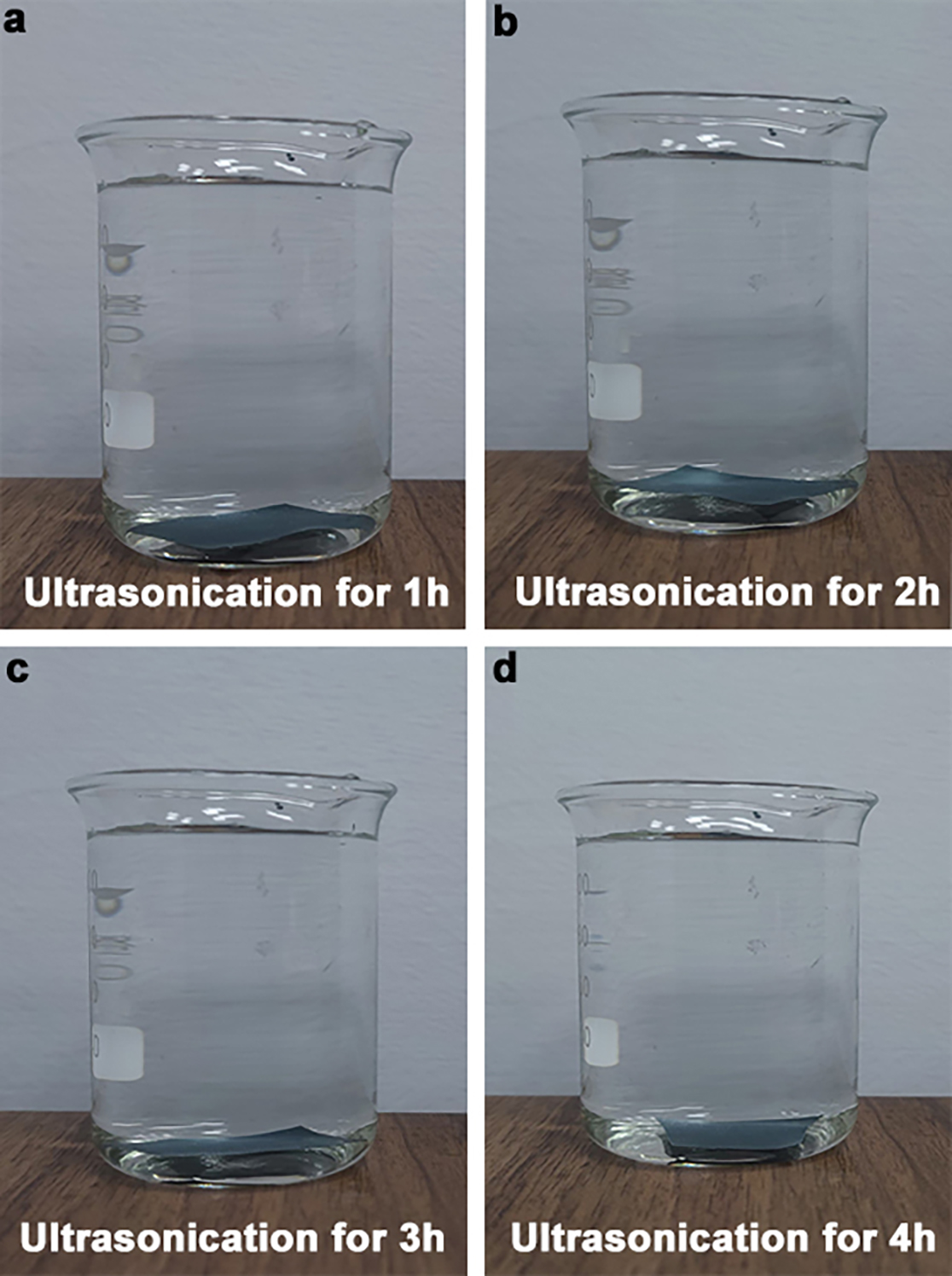


**Fig. S7** Photographs of the WPU/30FG@CuP@ZTC nanocomposite film during ultrasonication (using a 100 W, 40 kHz sonicator) in water

**
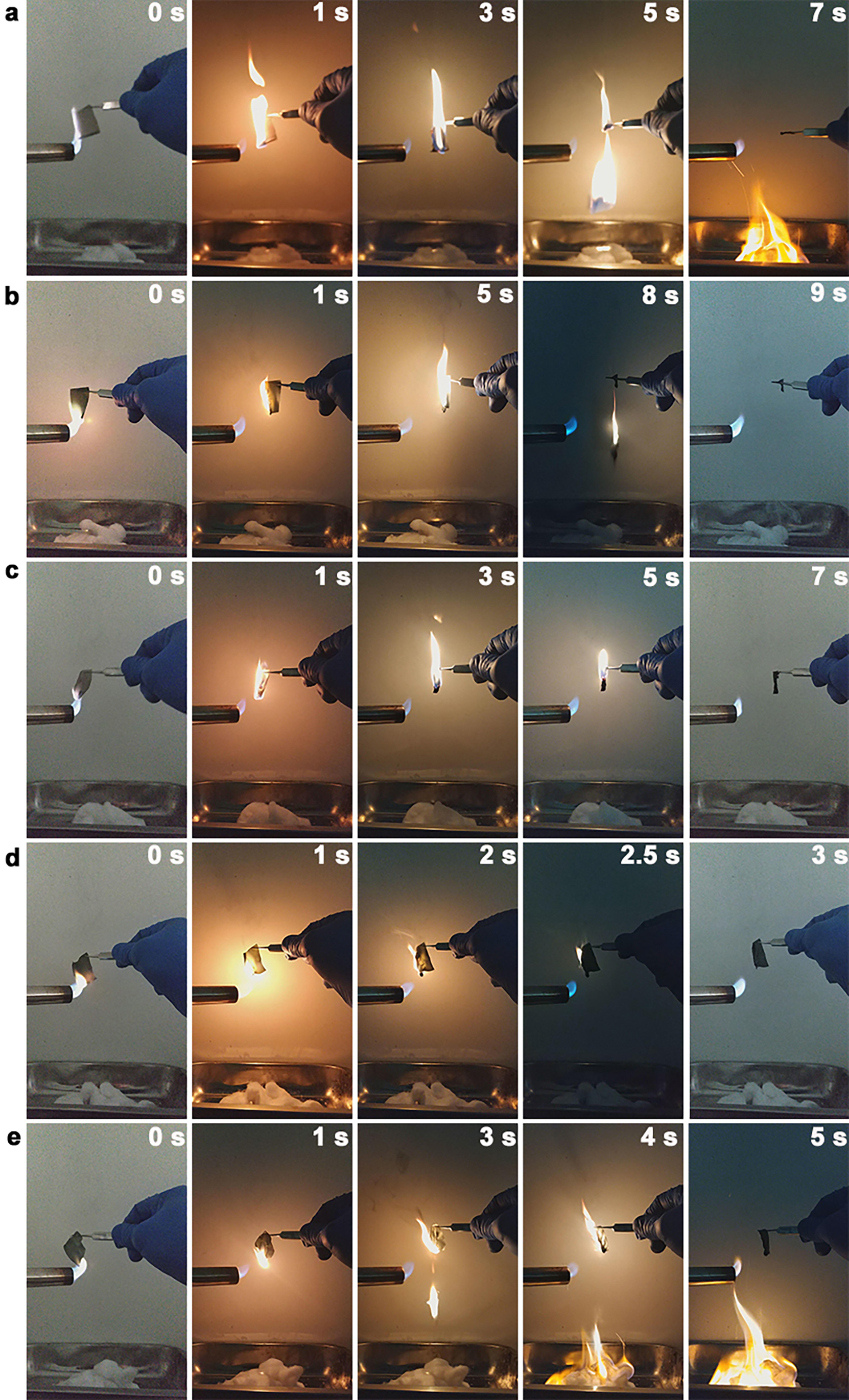
**

**Fig. S8** Digital photographs of **a** WPU/10FG@CuP@ZTC, **b** WPU/20FG@CuP@ZTC, **c** WPU/30FG/CuP/ZTC, **d** WPU/30FG@CuP, and **e** WPU/30FG@ZTC during flame burning test


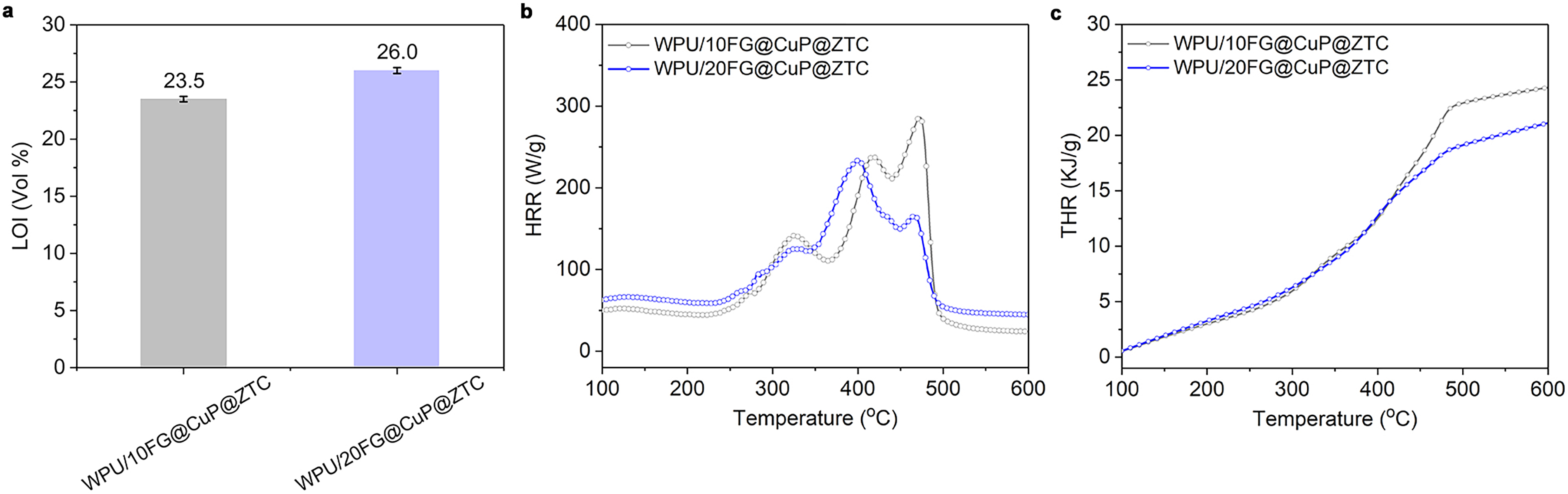


**Fig. S9** **a** LOI values, **b** HRR curves, and **c** THR curves for different WPU samples


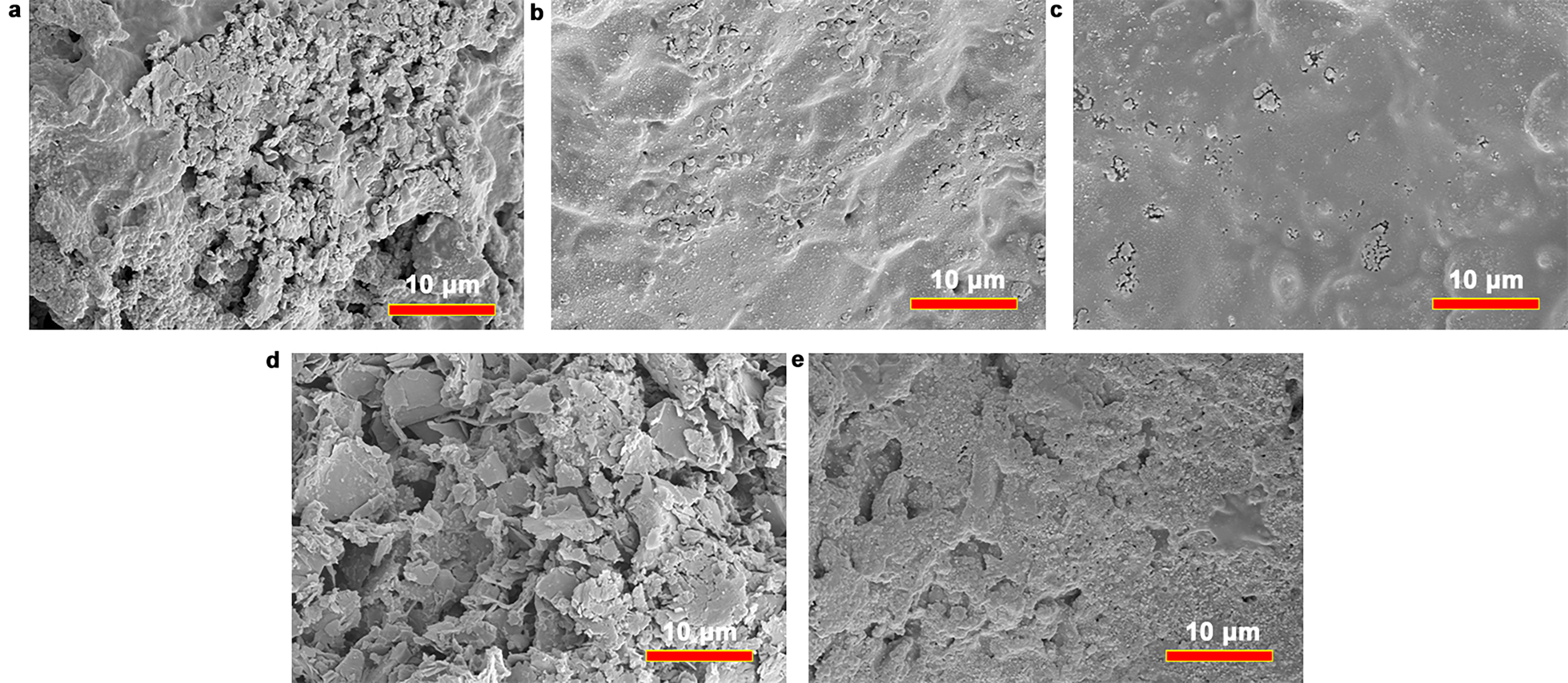


**Fig. S10** SEM images of char residues for **a** WPU/10FG@CuP@ZTC, **b** WPU/20FG@CuP@ZTC, **c** WPU/30FG@CuP, **d** WPU/30FG@ZTC, and **e** WPU/30FG/CuP/ZTC


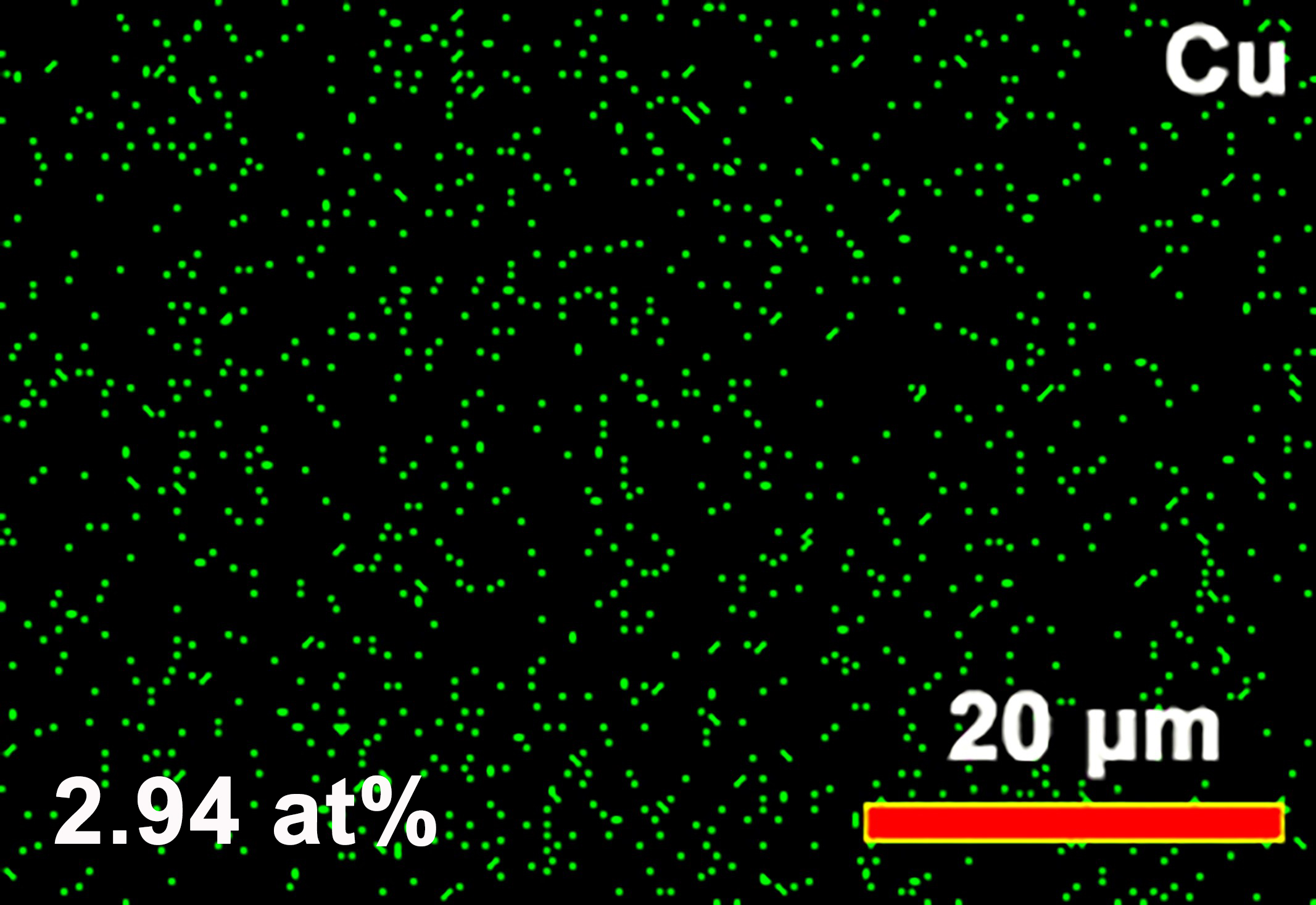


**Fig. S11** EDS mappings of WPU/30FG@CuP@ZTC


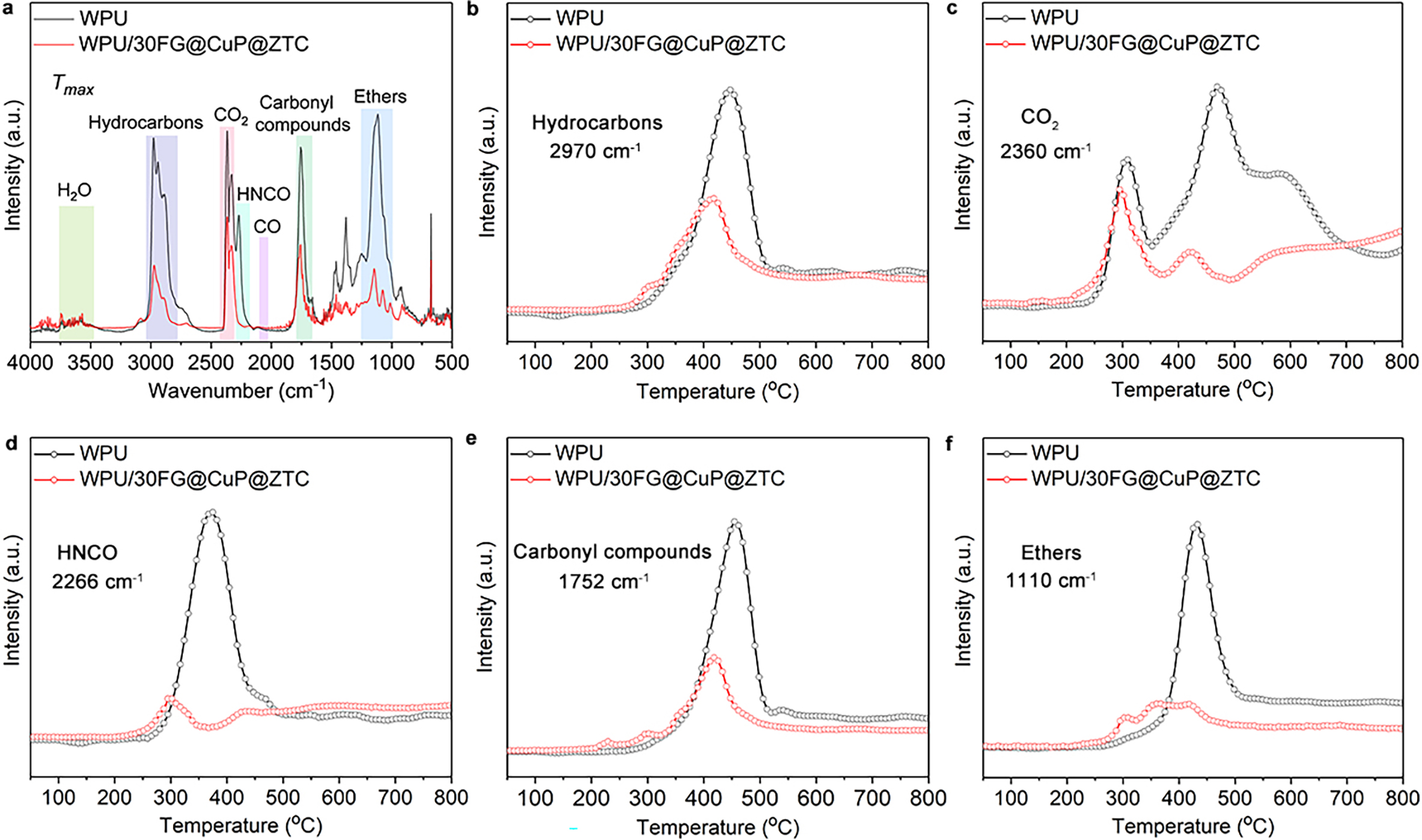


**Fig. S12** **a** FTIR spectra of pyrolysis products for pure WPU and WPU/30FG@CuP@ZTC at the temperature of the maximum loss rate. FTIR spectra of **b** hydrocarbons, **c** CO_2_, **d** HNCO, **e** carbonyl compounds, and **f** ethers for pure WPU and WPU/30FG@CuP@ZTC at different temperatures


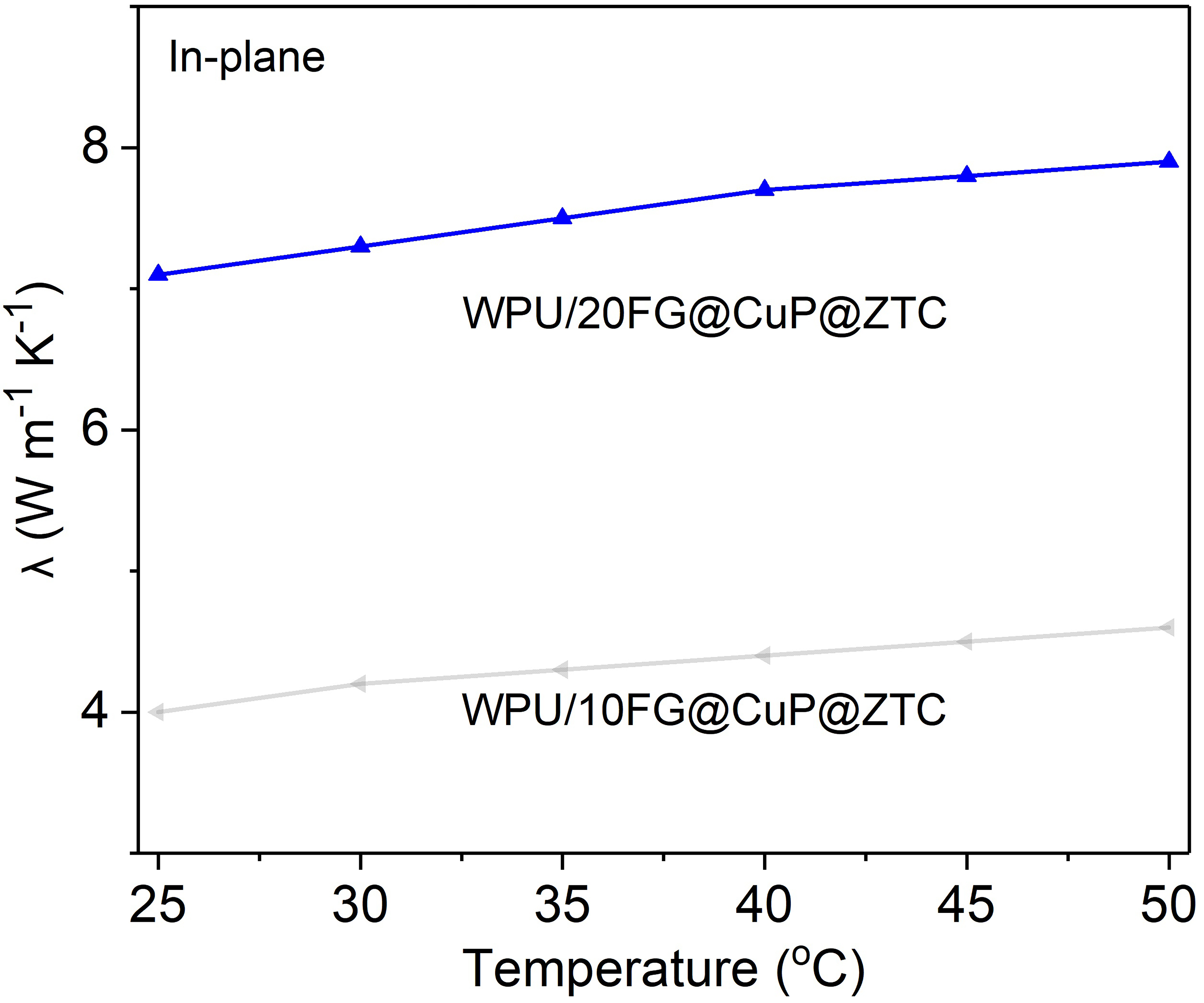


**Fig. S13** In-plane λ of different WPU samples as a function of temperature


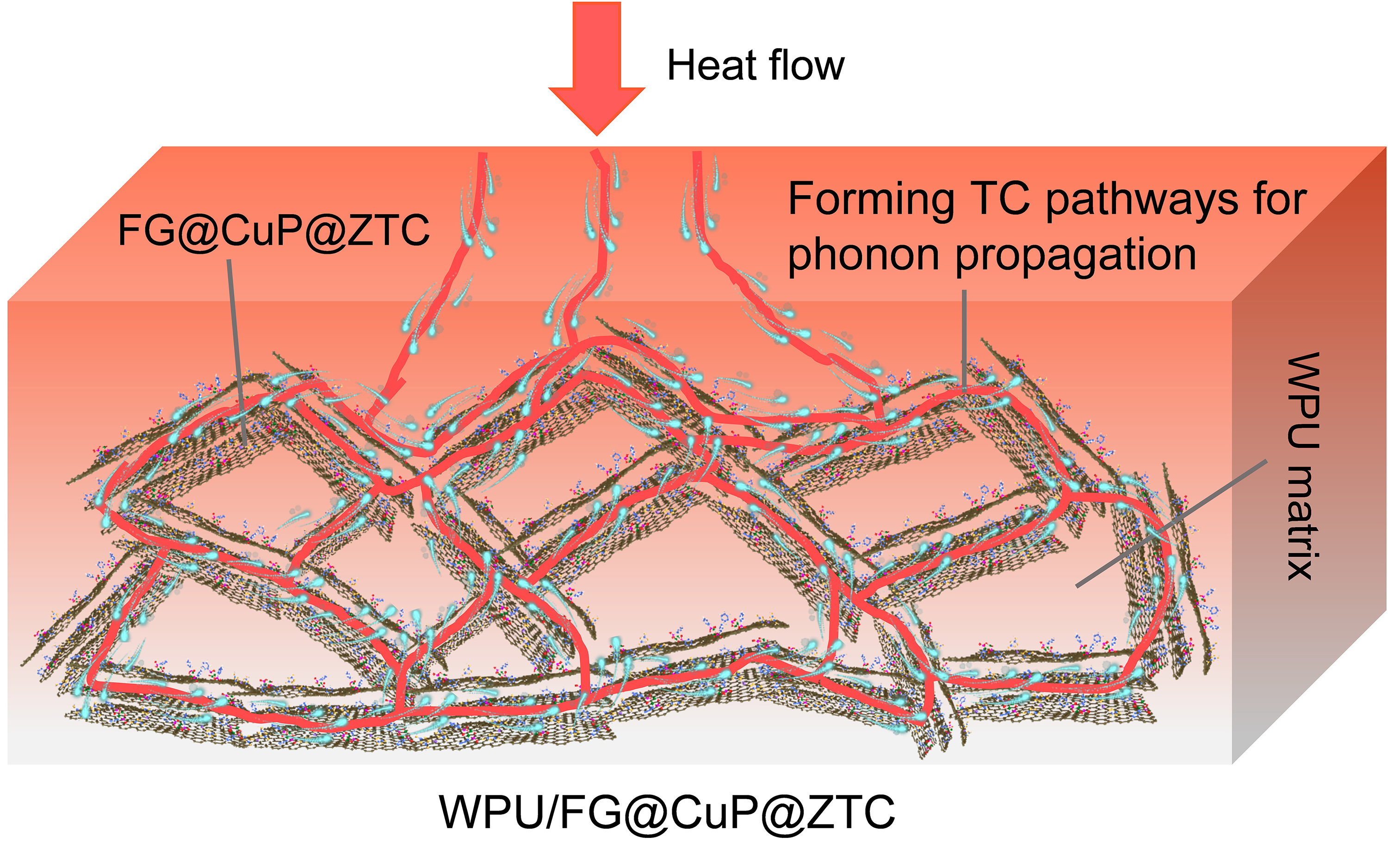


**Fig. S14** The schematic diagram of thermal conductive mechanism for WPU/FG@CuP@ZTC nanocomposite film


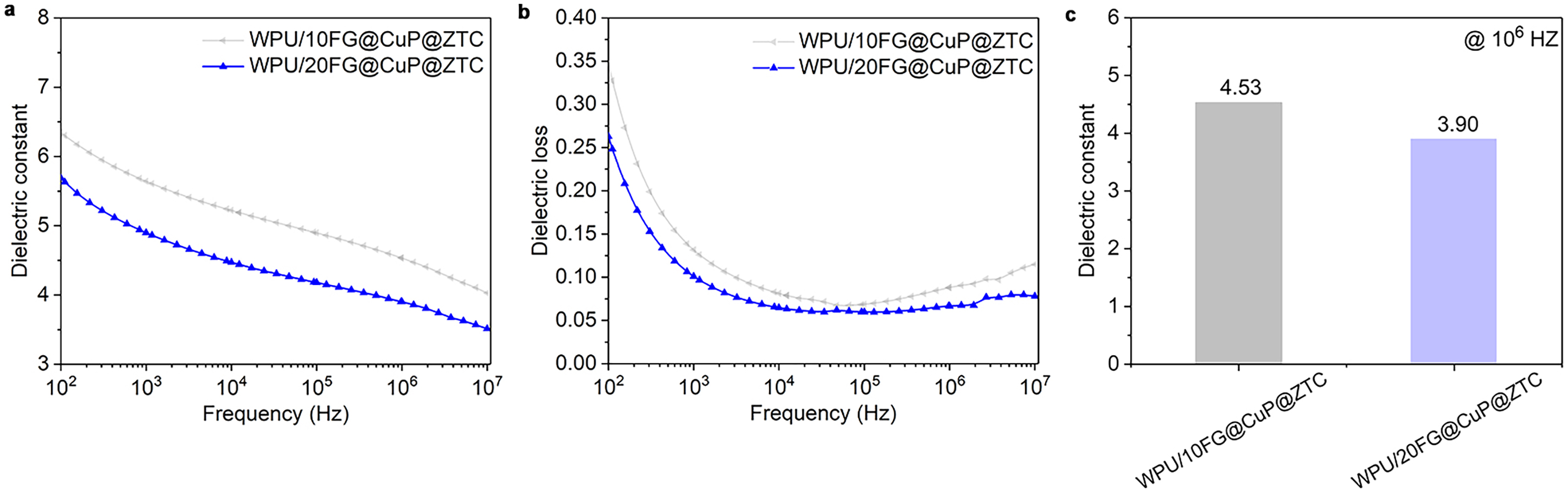


**Fig. S15** **a** Dielectric constant and **b** dielectric loss curves of different WPU samples. **c** dielectric constants of different WPU samples at 10^6^ Hz

**Table S1** Formulations of different WPU samples

| Sample code | WPU (wt%) | FG (wt%) | CuP (wt%) | ZTC (wt%) | FG@CuP^a^  (wt%) | FG@ZTC^b^ (wt%) | FG@CuP@ZTC^c^ (wt%) |
| --- | --- | --- | --- | --- | --- | --- | --- |
| WPU | 100 | - | - | - | - | - | - |
| WPU/10FG@CuP@ZTC | 90 | - | - | - | - | - | 10 |
| WPU/20FG@CuP@ZTC | 80 | - | - | - | - | - | 20 |
| WPU/30FG@CuP@ZTC | 70 | - | - | - | - | - | 30 |
| WPU/30FG/CuP/ZTC | 70 | 10 | 18 | 2 | - | - | - |
| WPU/30FG@CuP | 70 | 10 | - | - | 20 | - | - |
| WPU/30FG@ZTC | 70 | 10 | - | - | - | 20 | - |

FG@CuP^a^: a weight ratio of FG and CuP *ca*. 1: 2

FG@ZTC^b^: a weight ratio of FG and ZTC *ca*. 1: 2

FG@CuP@ZTC^c^: a weight ratio of FG, CuP, and ZTC *ca*. 1: 1.8: 0.2

**Table S2** Tensile strength and elongation at break of different WPU samples

| Sample | Tensile strength (MPa) | Tensile modulus  (MPa) | Toughness  (MJ/m^3^) | Elongation at break (%) |
| --- | --- | --- | --- | --- |
| WPU | 10.5 ± 0.8 | 24 ± 2 | 63 ± 4 | 858 ± 86 |
| WPU/10FG@CuP@ZTC | 13.7 ± 1.1 | 54 ± 4 | 60 ± 4 | 702 ± 70 |
| WPU/20FG@CuP@ZTC | 15.9 ± 1.3 | 68 ± 5 | 56 ± 3 | 546 ± 55 |
| WPU/30FG@CuP@ZTC | 20.3 ± 1.4 | 102 ± 7 | 45 ± 3 | 375 ± 38 |
| WPU/30FG/CuP/ZTC | 17.8 ± 1.3 | 72 ± 5 | 55 ± 4 | 493 ± 49 |

**Table S3** MCC test of pure WPU film and WPU composite films

| Sample code | PHRR (W/g) | THR (KJ/g) | EHC (KJ/g·K) | Char yield (%) |
| --- | --- | --- | --- | --- |
| WPU | 488 ± 24 | 27.9 ± 1.4 | 0.3712 ± 0.0186 | 0 |
| WPU/10FG@CuP@ZTC | 286 ± 14 | 24.3 ± 1.2 | 0.2259 ± 0.0113 | 7.5 |
| WPU/20FG@CuP@ZTC | 233 ± 12 | 21.1 ± 1.1 | 0.1942 ± 0.0097 | 12.4 |
| WPU/30FG@CuP@ZTC | 166 ± 8 | 16.6 ± 0.8 | 0.1475 ± 0.0073 | 21.2 |
| WPU/30FG/CuP/ZTC | 211 ± 11 | 19.5 ± 1.0 | 0.1795 ± 0.0090 | 15.6 |
| WPU/30FG@CuP | 194 ± 10 | 18.3 ± 0.9 | 0.1724 ± 0.0086 | 18.1 |
| WPU/30FG@ZTC | 339 ± 17 | 25.9 ± 1.3 | 0.2978 ± 0.0149 | 13.9 |

**Table S4** Quantitative assessment of the fire safety and main flame-retardant modes for WPU film and WPU composite films

| Sample | Flame inhibition (%) | Charring effect  (%) | Barrier and protective effect (%) |
| --- | --- | --- | --- |
| WPU | - | - | - |
| WPU/10FG@CuP@ZTC | 39.1 | 7.5 | 32.7 |
| WPU/20FG@CuP@ZTC | 47.7 | 12.4 | 36.9 |
| WPU/30FG@CuP@ZTC | 60.3 | 21.2 | 42.8 |
| WPU/30FG/CuP/ZTC | 51.6 | 15.6 | 38.1 |
| WPU/30FG@CuP | 53.6 | 18.1 | 39.4 |
| WPU/30FG@ZTC | 19.8 | 13.9 | 25.2 |
